# A parsimonious approach to predict regions affected by sewer-borne contaminants in urban aquifers

**DOI:** 10.1007/s10661-023-12027-6

**Published:** 2023-11-23

**Authors:** Karen L. Rojas-Gómez, Martin Binder, Marc Walther, Christian Engelmann

**Affiliations:** 1 Department of Aquatic Ecosystem Analysis, Helmholtz-Centre for Environmental Research – UFZ, Brückstraße 3a, 39114 Magdeburg, Germany; 2 Institute of Urban Water Management, Technische Universität Dresden, Bergstraße 66, 01069 Dresden, Germany; 3 Chair of Hydrogeology and Hydrochemistry, Institute of Geology, Technische Universität Bergakademie Freiberg, Gustav-Zeuner-Straße 12, 09599 Freiberg, Germany; 4 Department of Environmental Sciences, Applied and Environmental Geology, University of Basel, Bernoullistrasse 32, 4056 Basel, Switzerland; 5 Institute of Forest Growth and Forest Computer Sciences, Technische Universität Dresden, Pienner Straße 8, 01737 Tharandt, Germany

**Keywords:** Groundwater monitoring, Risk area identification, Sewer leakage, Sewer network, Transport simulation

## Abstract

Leaky urban drainage networks (UDNs) exfiltrating wastewater can contaminate aquifers. Detailed knowledge on spatiotemporal distributions of water-dissolved, sewer-borne contaminants in groundwater is essential to protect urban aquifers and to optimize monitoring systems. We evaluated the effect of UDN layouts on the spreading of sewer-borne contaminants in groundwater using a parsimonious approach. Due to the UDN’s long-term leakage behavior and the existence of non-degradable sewer-borne contaminants (equivalent to a conservative and constant contaminant source), we employed a concept of horizontal line sources to mimic the UDN layout. This does not require the consideration of bio-degradation processes or temporal delay and effectively bypasses the vadose zone, thus reducing computational requirements associated with a full simulation of leakages. We used a set of synthetic leakage scenarios which were generated using fractals and are based on a real-world UDN layout. We investigated the effects of typical leakage rates, varying groundwater flow directions, and UDN’s layouts on the shape of the contaminant plume, disregarding the resulted concentration. Leakage rates showed minimal effects on the total covered plume area, whereas 89% of the variance of the plume’s geometry is explained by both the UDN’s layout (e.g., length and level of complexity) and groundwater flow direction. We demonstrated the potential of applying this approach to identify possible locations of groundwater observation wells using a real UDN layout. This straightforward and parsimonious method can serve as an initial step to strategically identify optimal monitoring systems locations within urban aquifers, and to improve sewer asset management at city scale.

**Supplementary Information:**

The online version contains supplementary material available at 10.1007/s10661-023-12027-6.

## Introduction

By 2050, cities are expected to concentrate up to 70% of the global population (UNESCO, 2019), 30% more than the current urbanization level. This rapid urbanization process creates various challenges especially for managing groundwater resources. An increasingly negative impact on groundwater availability, simultaneously affecting raw water quality and quantity (Schirmer et al., 2013), is one of these challenges. For instance, urban drainage networks (UDNs), located partially or completely above the groundwater level and conducting untreated wastewater to municipal and/or decentral wastewater treatment plants, may have failures leading to unintended exfiltration. That means, when the hydraulic potential is larger in the sewer pipe than in the surrounding (partially) water-saturated zone, wastewater may leak into the vadose zone and groundwater bodies (Ellis et al., 2003; Nguyen et al., 2021; Vystavna et al., 2018). Increasing UDN age can generate a progressive deterioration, with failures and local leakages (Ellis & Revitt, 2002). As a rule of thumb, exfiltration rates may reach up to one-tenth of the average daily dry weather flow, i.e., the base wastewater flow in sewer infrastructure (Ellis et al., 2003). Among various sewer-borne contaminants, pathogens can bear health risks for humans (Ellis & Revitt, 2002; Held et al., 2007; Reynolds & Barrett, 2003) and nutrients may affect groundwater ecosystems by changing the chemical and microbial conditions therein (Erostate et al., 2018; Haßler et al., 2018). Emerging contaminants, such as pharmaceuticals, drugs, personal care products, or disinfection by-products, were found in multiple aquifers. It is assumed that such contamination patterns are a consequence of sewer leakages, especially for urban shallow aquifers (Jurado et al., 2012; Lee et al., 2015; Roehrdanz et al., 2017; Rusiniak et al., 2021). These constituents may be potentially persistent, mutagenic, and toxic. Their combination may further trigger synergistic effects, increasing their negative impacts even at very low concentrations (Barouki, 2017; Pamplona-Silva et al., 2018).

The spatiotemporal distribution of such contaminants in a specific urban catchment depends on (i) hydrological and climatological characteristics (e.g., precipitation intensities and patterns); (ii) hydrogeological settings, in particular directly beneath the sewer pipes (e.g., hydraulic permeabilities); (iii) chemical properties of the pollutants, in particular their reactivity with the natural materials (e.g., sorption coefficients); (iv) past and present urban land use (e.g., surface sealing above sewer pipes); and eventually (v) the layout of the (potential) sources of contamination, i.e., leaky sewers (Schirmer et al., 2013; Tubau et al., 2017; Wolf et al., 2004).

Given the harmfulness of contaminants, in order to support a sustainable urban water management, the identification of sewer leakages and corresponding water-dissolved contaminant plumes in aquifers have gained increasing interest (Grimmeisen et al., 2017; Kobayashi et al., 2021; Nikolenko et al., 2023; Panasiuk et al., 2015; Verovšek et al., 2023; Vystavna et al., 2018). Various physical as well as numerical flow and transport models have been developed and applied, ranging from laboratory up to city scale to understand and forecast the water distribution within UDNs as well as the potential for either exfiltration or infiltration (e.g., Karpf & Krebs, 2011; Rutsch et al., 2008; Wolf et al., 2007). However, the quantitative relationship between the UDN’s layout and the potential spreading of sewer-borne contaminants in urban aquifers has received far less attention. One reason for this may be the complexity of real-world sewer networks and urban settings in general, requiring a rather detailed description of the positions and characteristics of leakage areas as well as sophisticated inspection methods, which are costly and time-consuming (Sadeghikhah et al., 2022). Such inspection methods usually bear significant levels of uncertainty, among others attributed to the subjectivity of the operators (Caradot et al., 2020; Roghani et al., 2019). Additionally, the evaluation of external conditions around a sewer segment is technically unfeasible while employing such internal surveillance techniques. The verification of each contaminant source separately (i.e., each pipe failure) is, therefore, difficult to unfeasible considering the total length of such UDNs (typically a few hundreds of kilometers) and the large spatial variation of such failures within them. Furthermore, detailed underground infrastructure data is often unavailable or confidential (Blumensaat et al., 2012). Water utilities and/or sewer operators face significant challenges related to sewer asset data management, affecting data availability, quality, and capability of data processing (Tscheikner-Gratl et al., 2019). However, it has been demonstrated before that combining rather fundamental data (among others: approximations of sewer locations based on road courses, groundwater levels and flow directions, climate data, land use maps, soil, and hydrogeological maps) with standard numerical modeling may be sufficient for detecting leaky sewer segments (Lee et al., 2015; Roehrdanz et al., 2017; Sadeghikhah et al., 2022).

In this article, we use the principle of parsimony to model and to assess the propagation of sewer-borne contaminants in urban aquifers. We hypothesize that the long-term spreading of solutes originating from leaky sewer pipes is closely correlated to the UDN’s layout, i.e., their spatial arrangement in the horizontal plane. We furthermore assume that, on a long-term perspective, the percolation of sewage through the vadose zone is primarily vertical in consequence of gravity. Hence, UDNs subject to exfiltration can be mimicked using horizontal line sources of contamination (HLSCs) located directly at the groundwater table. We used numerical flow and transport modeling in junction with GIS-aided geoprocessing tools to delineate potentially polluted regions in urban aquifers considering long-term contamination associated with sewer leakages. Here, the sewer leakages were represented (i) as synthetically generated HLSCs following fractal geometries (Ghosh et al., 2006) and (ii) an actual UDN shape of an exemplary urban area.

## Materials and methods

We simulated 288 scenarios with different HLSCs using numerical groundwater flow (MODFLOW-2005; Harbaugh, 2005) and solute transport modeling (MT3D-MS; Zheng, 2010). The variations were generated using a fractal geometry approach, with an increasing complexity of the HLSC layouts. A real-world sewer network layout was modeled in addition. The leaky HLSC layouts were defined as Neumann-type boundary conditions and the generated contaminant plumes were evaluated delineating risk areas of elevated concentrations. A set of geoprocessing and spatial analysis tools implemented in QGIS 3.4 (OSGeo, 2019) such as the Delaunay triangulation, dissolve geoprocessing tool, and intersection of polygons were employed to evaluate the relationship between the UDN’s layout and the contaminant plume in the fully water-saturated groundwater zone. The Delaunay triangulation allowed the creation of polygons to represent both the HLSC covering drainage areas and the plume areas, whereas the other vector geometry tools facilitated the computation of geometric characteristics (i.e., area, geometrical centroid, coordinates).

### Conceptional model: HLSC as a parsimonious representation of UDN exfiltration

The spreading of contaminants in aquifers originating from leaky sewer networks is associated with contaminant transport through the backfill trench material surrounding the pipe, the vadose zone, and, eventually, in the fully water-saturated saturated aquifer. Exfiltration in the near vicinity of the pipes is governed by the presence of a colmation layer, i.e., a transition zone formed between the pipe and the surrounding backfill material (Ellis et al., 2003; Nikpay, 2015). The hydraulic properties of these colmation layers may differ strongly between sanitary and stormwater sewer networks due to differences in the water compositions and solid contents (Peche et al., 2019). Exfiltration becomes a steady-state process, with constant exfiltration rates once the hydraulic characteristics of the colmation layer remain temporally invariant (Karpf, 2012). Due to this simplification, assuming a constant and stationary water flow through the colmation layer, it is possible to reduce the actual three-dimensional sewer exfiltration dynamics to one spatial dimension in vertical direction (i.e., *z* coordinate; Nguyen et al., 2021). The relatively high-permeable backfill trenches contribute to a preferentially horizontal distribution of solutes along the pipe trenches (“urban karst” concept; e.g., Bonneau et al., 2017; Kaushal & Belt, 2012). Considering this, the HLSC would horizontally follow the pipe segment and, consequently, the UDN’s layout. Once sewage passes the bedding material, contaminant percolation towards the groundwater table is then primarily occurring in vertical direction due to gravitational force (Tubau et al., 2017). This means, as a simplification, the UDN’s layout can be projected as a contaminant source at the groundwater surface, mimicking the resulting exfiltration into the groundwater. This reduces the three-dimensional system to a two-dimensional system (i.e., *x*-*y* plane), considerably reducing computational efforts for the simulation. Therefore, the UDN’s layout (e.g., length of sewer pipes, intersection angles) may eventually influence the geometry of contaminant plumes in groundwater. Please note that this projection concept considers that the leakage rate is independent from the soil water pressure beneath the pipe defect and groundwater levels (Peche et al., 2019).

### Base case numerical groundwater flow and solute transport model

**Fig. 1:**
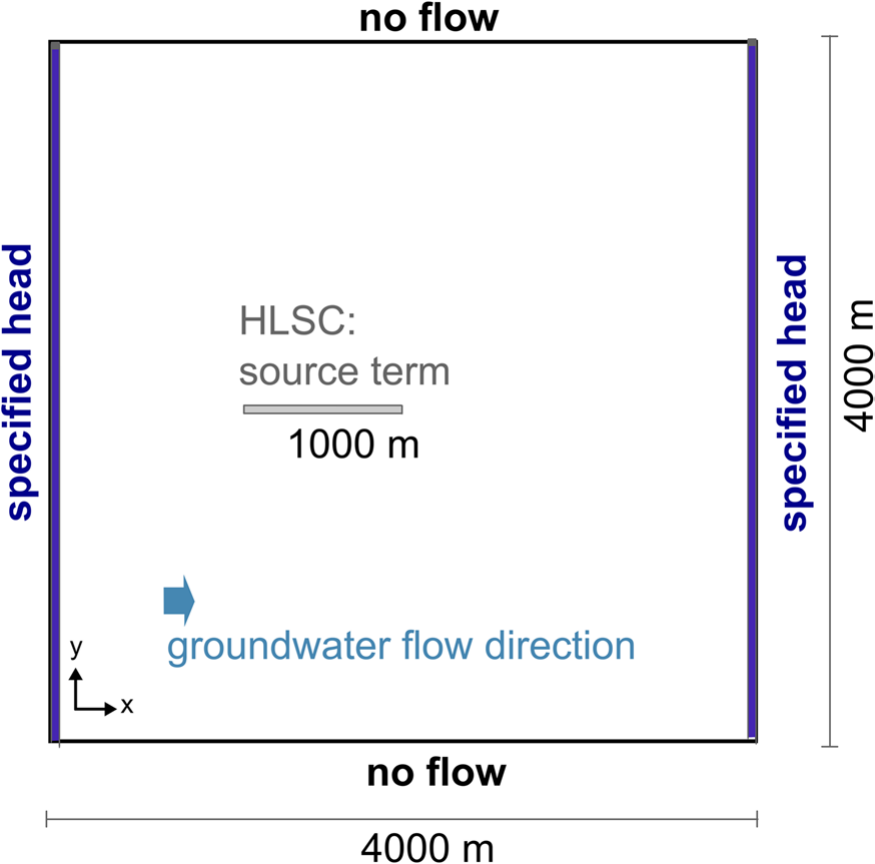
Model setup for groundwater flow and transport including boundary condition definition and illustration of the base case scenario of UDN exfiltration (single main pipe, indicated with gray line of 1000 m length that represented a HLSC simulated as source term)

The model setup, mimicking the transport of an inert solute in an unconfined, homogeneous, and shallow aquifer, is summarized in Table 1 and illustrated in Fig. 1. The hydraulic gradient was defined by assigning specified head boundary conditions (1st type, i.e., Dirichlet-type) with time-invariant values to the opposite sides of the model. The uniform simulation time of 10 years was iteratively selected based on a set of preliminary test runs (not shown). All model setups (pre and main runs) reached its final state at this simulation time and longer times did not show a significant effect on the relative change of the shape of contaminant plumes. Additionally, for the given aquifer characteristics and hydraulic gradient, the selected leakage rates do not significantly change the groundwater flow direction. This study does not consider the possible influence of different lithologies on the flow rates and plume evolution. The systematic definition and analysis of UDN layouts as well as data processing was implemented in Python (PSF, 2018). The FloPy package (Bakker et al., 2016) was used for creating model input files, running simulations, and reading model output data generated by MODFLOW and MT3D-MS. This workflow was carried out for the base case scenario as well as for all variation scenarios.

**Table 1:** Hydraulic characteristics of the aquifer simulated. Values for hydraulic conductivity, porosity, and diffusion coefficient were taken from Bear and Cheng (2010)

Parameter	Setting/value
*Spatial model settings*	
Horizontal length of the model area in *x* direction	4000 m
Horizontal length of the model area in *y* direction	4000 m
Thickness of the aquifer in *z* direction	10 m
Size of the regular mesh grid (*x* × *y* × *z*)	5 m × 5 m × 10 m
Number of cells in the horizontal plane (*x* × *y*)	800 × 800
Number of model layers (*z*)	1
*Temporal model settings*	
Total simulation time	10 years
Flow simulation	Steady state, flow in *x* direction
Solute transport simulation	Steady state, conservative (advection, dispersion, and diffusion are simulated)
*Aquifer properties*	
Type	Unconfined
Sediment type	Sand
Fully water-saturated horizontal hydraulic conductivity *K*_*H*_	1 × 10^−4^ m s^−1^
Hydraulic gradient *i*	0.002 m m^−1^
Effective porosity *n*	0.3
Pore diffusion coefficient *D*_*P*_	5 × 10^−10^ m s^−2^
Longitudinal horizontal dispersivity *α*_*L*_	1 m
Transversal horizontal dispersivity *α*_*TH*_	0.1 m
*Properties of the HLSC (base case scenario (BCS))*	
Length of pipe	1000 m (i.e., mimicking a juxtaposition of multiple smaller pipe segments)
Realization of water leakage	Positive 2nd-type boundary condition, i.e., Neumann-type; flux rate of 2·10^−8^ m^3^ s^−1^ per grid cell
Injected mass flux at the leakage location	Depending on leakage rate mass varies between 2·10^−8^ and 2·10^−6^ kg s^−1^ per grid cell. This assumes a solute concentration of 1 kg m^−3^ via a source term

### Definition of synthetic scenarios based on the fractal geometry approach

#### UDN layouts

**Fig. 2:**
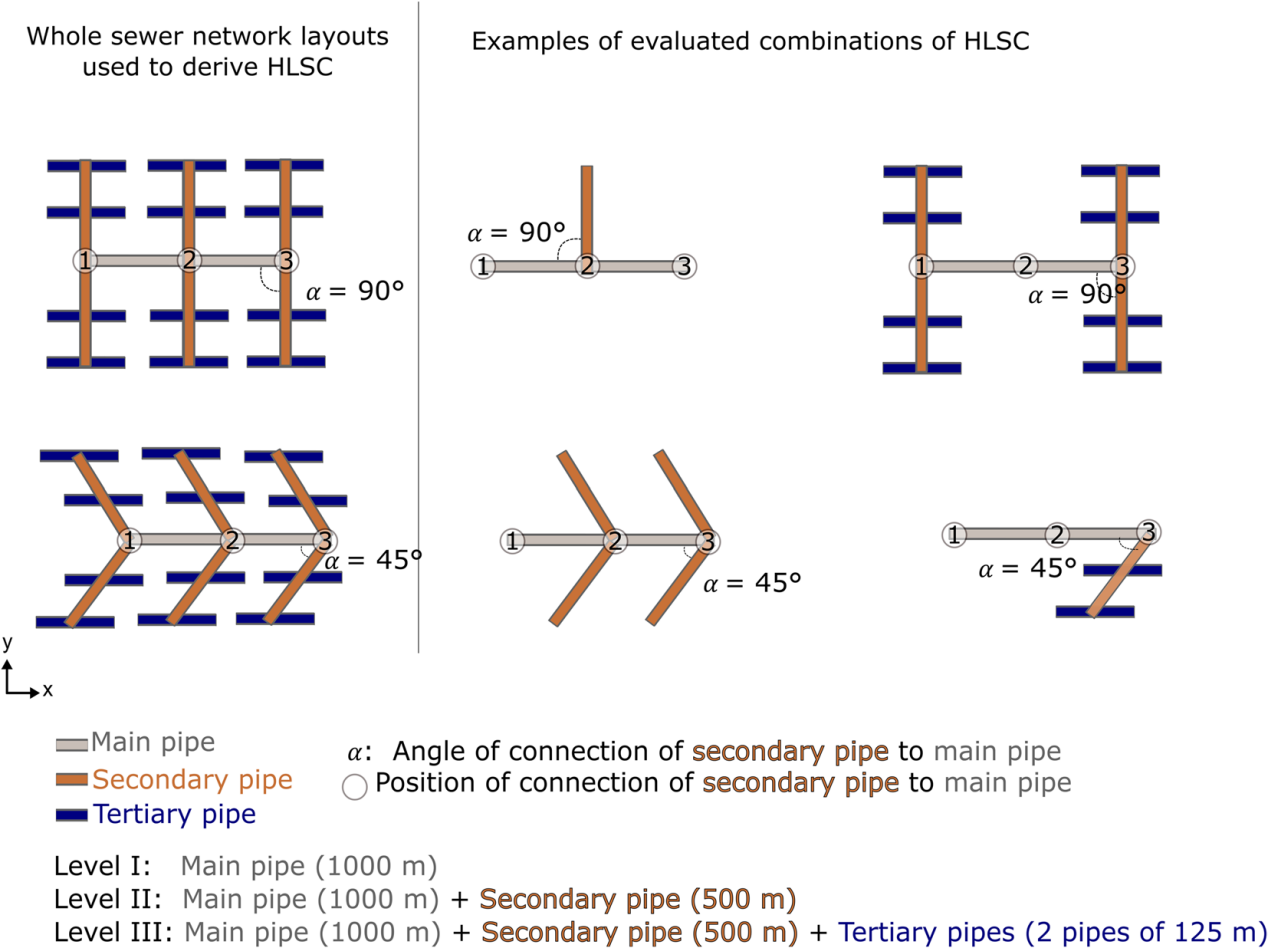
Examples of sewer network layouts used for defining HLSC scenarios with three levels of complexity (left) and further examples of HLSC combinations (right). Depending on the level of complexity (I to III), the main pipe and secondary/tertiary pipes were added. The characteristics of the main pipe (gray line) remained constant for all the scenarios, whereas the number of secondary/tertiary pipes (brown/dark blue) was varied. Variations were also carried out for the position (circles 1, 2, and 3) and intersection angles of the secondary pipes to the main pipe

The influence of the UDN’s structural properties was evaluated starting with network layouts of straightforward and intentionally simple structure (e.g., a single pipe of a specific length), followed by more complex ones generated by implementing a fractal geometry approach after Ghosh et al. (2006). All these layouts included junctions and conduits (Fig. 2), which correspond to nodes and links draining to a single outlet (Yang et al., 2017). The UDN layouts generated included single and multiple sewer pipe segments with different horizontal alignments, i.e., up to three positions of connections in junctions 1, 2, and 3 along the main pipe (see right part of Fig. 2) and varying intersection angles (left part of Fig. 2). A hierarchy of pipe diameters and lengths is defined, thus mimicking main, secondary, and tertiary pipes to consider different levels of UDN complexity (Table 2). Overall, 36 different layouts were designed. More sophisticated approaches to generate realistic UDN incorporating specific spatial characteristics of a given study area have been proposed (Blumensaat et al., 2012; Reyes-Silva et al., 2023). However, these approaches are too laborious to be applied at this first stage of the investigation. Hence, we used the space-filling method of fractal tree geometries (Ghosh et al., 2006), which is a straightforward and parsimonious approach to generate synthetic sewer networks. This allows to quantify the relationship between the UDN’s layout and the spreading of sewer-borne contaminants in urban aquifers in a systematic way.

**Table 2:** Characteristics of the levels of complexity (LC) for the artificial sewer networks

Level	Characteristics	Length of pipe per LC	Description	*L*_HLSC_
I	1 main pipe	1000 m	Main pipe only	1000 m
II	1 main pipe +	1000 m	Secondary pipes are connected to the main pipe either at one side or both sides of the main pipe	1500 to 4000 m
	1 to 6 secondary pipe(s)	500 m		
III	1 main pipe +	1000 m	Tertiary pipes are connected to the secondary pipe at each side of the pipe	1750 to 7000 m
	1 to 6 secondary pipe(s) +	500 m		
	2 to 24 secondary pipe(s)	125 m		

#### Hydraulic leakage rates of the UDNs

For each UDN layout created, two hydraulic leakage rate scenarios were analyzed (Table 3). The first leakage scenario included a time-invariant and uniform leakage rate assigned to all HLSCs without considering the hierarchy of the pipes. Contrary, the second leakage scenario considered a different leakage rate per HLSC type. For example, the shortest HLSC (tertiary pipe) can represent pipes of less frequent maintenance, small diameter, and shallow burial depth (e.g., household connections to the municipal sewer network). These tertiary pipes were assumed to be more exposed to deterioration and may have highest exfiltration rates (Caradot et al., 2018; Reynolds & Barrett, 2003). In contrast, assuming more maintenance frequency and easier accessibility, main pipes would have the lowest leakage rate. The leakage rate for each HLSC type (Table 3) was selected considering typical values of exfiltration based on real-world sewer networks reported by previous studies (Ellis et al., 2003; Karpf, 2012; Karpf & Krebs, 2011; Nguyen & Venohr, 2021; Peche et al., 2017). The value of leakage rate integrates the impact of the clogging layer and influence of the hydraulic properties of the trench backfill material (Karpf & Krebs, 2011). Eventually, two groundwater flow directions (GWD) were evaluated. The first scenario considered flow along the *x* direction, whereas the second scenario considered flow along the *y* direction. The groundwater flow was driven by the difference in constant heads in both cases. No natural recharge was considered. In total, 288 scenarios were defined and simulated.

**Table 3:** Evaluated scenarios considering 36 different basic HLSC layouts (i.e., combinations of pipes) at the 2nd and 3rd level of complexity (LC), with two options of pipe intersection angles (*α*), leakage rate (LR), and groundwater flow direction (GWD) each. In total, 288 scenarios were simulated (36 layouts × 2 angle options × 2 leakage scenarios × 2 groundwater flow directions)

Scenario variation	Description of options	Combinations
Basic HLSC layouts	3 levels of complexity (LC) were implemented, as shown in Table 2. For more details including a listing of the layouts, please refer to Tables S1–S2 and Figures S1–S2.	36
Angles of intersections (*α*)	Both (i) 90° and (ii) 45° were realized. Hereby, the alignment was realized as shown in Fig. 2, i.e., main and tertiary pipes remain in parallel to each other.	2
Leakage rate (LR) scenarios	Two scenarios (i) and (ii) with uniform and non-uniform leakage rates were realized, respectively: (i) Uniform distribution (1 × 10^−7^ m^3^ s^−1^ m^−1^) (ii) Variable setting for each type of pipe (main: 1 × 10^−8^ m^3^ s^−1^ m^−1^; secondary: 1 × 10^−7^ m^3^ s^−1^ m^−1^; tertiary: 1 × 10^−6^ m^3^ s^−1^ m^−1^)	2
Groundwater flow direction (GWD)	Groundwater flow was defined (i) along the *x*-axis and (ii) along the *y*-axis. This indirectly reflects turning the UDN layout by 90°.	2

### Using a real-world UDN layout to delineate risk areas

**Fig. 3:**
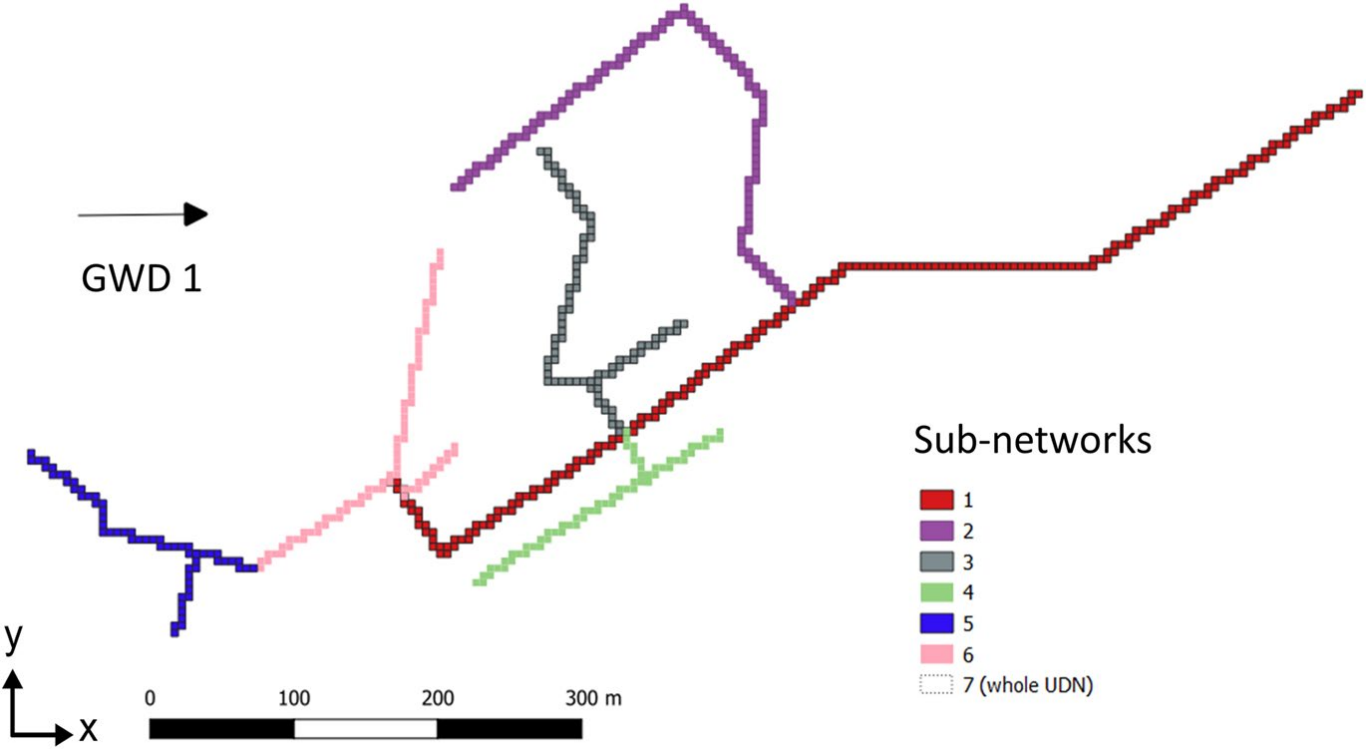
Geometrical representation of a small part of a UDN of Dresden, Germany, serving as non-synthetic reference to exemplify the application of the parsimonious method. Aquifer characteristics were taken from the BCS (Table 1); leakage rates were equal for all subnetworks

In general, each part of an UDN can be simulated as an individual HLSC. Each HLSC then generates a contaminant plume with a different shape. Considering steady-state flow conditions and a long-term contaminant transport, these contaminant plumes may overlap. The overlapping of the plumes would generate areas with higher mixture of solutes. The mixture of contaminants (e.g., endocrine disruptors) may have additive or synergistic effects leading to higher environmental risks (Barouki, 2017). We assumed that the probability of pollution due to a leaky pipe correlates with the number of intersections of contaminant plumes in a given area of the aquifer (in other words, more plume intersection equals higher risks). Hence, using our parsimonious approach, one can simulate contaminant plumes generated by individual HLSC in independent model runs, intersect those individual plumes, and identify areas with high pollution. A real-world UDN layout was used to exemplarily identify areas of potential risk for contamination and, based on that, to recommend point-based monitoring locations (typically given by groundwater observation wells). A part of a sewer network of the city of Dresden, Germany, served as a template to generate a more realistic UDN layout (Fig. 3). The UDN of choice covers a total length of 2910 m. The simulated scenarios included different HLSC combinations with a constant leakage rate of 1 × 10^−7^ m^3^ s^−1^ per m (Karpf & Krebs, 2011). Seven different HLSC were simulated (subnetworks 1 to 6 in Fig. 3) including the entire network (subnetwork 7 in Fig. 3). The subdivision of the system considered pipelines of similar diameters located in pre-defined branches of the UDN. In general, the HLSCs range from a LC I to III, having a minimum and maximum individual length (*L*_HLSC_) of 330 m and 2910 m, respectively (details in Table S3). Due to the generic modeling approach employed in this study, no local hydrogeological conditions such as physical heterogeneities were considered. In particular, the aquifer characteristics were assumed to be equal to the fully synthetic base case scenarios (BCS; see Table 1). The network layout was used as an exemplary, non-synthetic UDN, with groundwater flow direction and leakage rates kept the same as for the previous synthetic scenarios.

### Evaluation of factors influencing the spreading of a contaminant plume

Different HLSCs will release a different contaminant mass into the aquifer. The contaminant concentration inside a contamination plume will vary covering different orders of magnitude, whereby the edges of the plume show the smallest contaminant concentration. However, long-term plume response of emerging contaminants may affect human health and ecosystems even at very low concentrations (Barouki, 2017). Hence, the analysis focused on the shape of the entire plume generated by each HLSC, but not on the concentration distribution inside the plumes. Therefore, as a simplification, the edge of the plume represented the limit to calculate the total area under risk of pollution, since it comprises all possible regions affected by contamination. A relative threshold value for the contaminant concentration within the aquifer was defined to compute the area of the plume. This threshold considered a fraction of the maximum contaminant concentration in the aquifer for a given scenario, with

1 $${E}_{\textrm{plume}}=\frac{\operatorname{Max}\ (C)}{F_{c, th}}.$$

Hereby, Max (*C*) (kg m^–3^) is the maximum contaminant concentration observed within the model domain typically associated with a given UDN. Nevertheless, this variable may not be directly equivalent to the concentration of the “labeled” water that is leaky from the UDN. This is because the water may become diluted by mixing with non-labeled groundwater. *E*_plume_ is the minimum concentration limit to define the edge of the contaminant plume based on a user-defined absolute concentration threshold factor *F*_*c*, *th*_ (-). In this study, the value 10^9^ was chosen for *F*_*c*, *th*_. That means that if the maximum contaminant concentration observed in the model aquifer equals, for instance, 1 kg m^–3^, the edge of the plume would have a concentration of 10^−9^ kg m^–3^ (representing a magnitude of picogram per liter). The plumes were characterized considering their total spreading area (*A*_*p*_). In order to facilitate the comparison among all scenarios in this study (Table 3), values of *A*_*p*_ for each scenario *j* were compared to the base case scenario by

2 $${\textrm{RA}}_j=\frac{A_{p,\textrm{SC}}}{A_{p,\textrm{BCS}}}.$$

Here, RA_*j*_ (-) is the relative area of the contaminant plume for scenario SC, *A*_*p*, SC_ (m^2^) is the total area of the contaminant plume for scenario SC, and *A*_*p*, BCS_ (m^2^) is the total area of the contaminant plume for the base case scenario.

A multivariate statistical analysis was carried out using the *R* software code (R Core Team, 2016) in order to quantify the impact of the HLSC’s characteristics (Table 2) on the contaminant plume area *A*_*p*_ and the relative value for RA_*j*_. For this, the *R* packages “stats” (included in *R*), “car” (Fox & Weisberg, 2019), and “pastecs” (Grosjean et al., 2018) were used. Among others, a Pearson correlation matrix was computed to identify factors showing high correlation. An analysis of variance (ANOVA) was carried out to identify those factors (i.e., geometry of HLSCs, GWD, and LR) having a statistically significant effect on contaminant spreading.

## Results and discussion

### Using fractal UDN layouts to analyze contaminant spreading (synthetic scenarios)

#### Variations of HLSC layouts and hydraulic conditions

A total of 78 fractal UDN layouts and, hence, HLSC scenarios were simulated (Tables 2 and 3; details in Tables S1–S2 and Figures S1–S2). This included all levels of complexity I (6 layouts) as well as II and III (72 layouts). For LC I, the effective width of the HLSCs varied between 5 and 500 m, and the single HLSC was rotated (45°, 90°, and 135°). The HLSCs covered drainage areas between 0.17 and 1.24 km^2^, with a total UDN length ranging from 1000 m (base case scenario (BCS)) up to 7000 m (setting with maximum value for *n*_pipes_). In the case of LC I, the shape of the contaminant plume and RA_*j*_ change depending on the width of the HLSC and the angle formed by HLSC and the groundwater velocity vectors. Increasing the width of the HLSC from 5 to 500 m increases RA_*j*_ up to three times (from 1 to 3.4). HLSCs having widths larger than 5 m represented areas of leakages instead of the actual structures of real sewer networks. Hence, no other scenarios were derived considering different HLSC widths. A single HLSC parallel to the GWD (as in BSC) generates the smallest spreading of contaminants compared to a HLSC with the same length (1000 m) forming angles of 45°, 90°, and 135° with the GWD. HLSCs positioned perpendicular to the groundwater velocity vectors have a larger spreading of contaminants towards the flow gradient, reaching values of RA_*j*_ equal to 2.3. Only in case of single HLSCs having supplementary angles (angles that sum 180°, e.g., 45° and 135°), the area of the contaminant plume remains the same (details in Tables S4–S5).

**Fig. 4:**
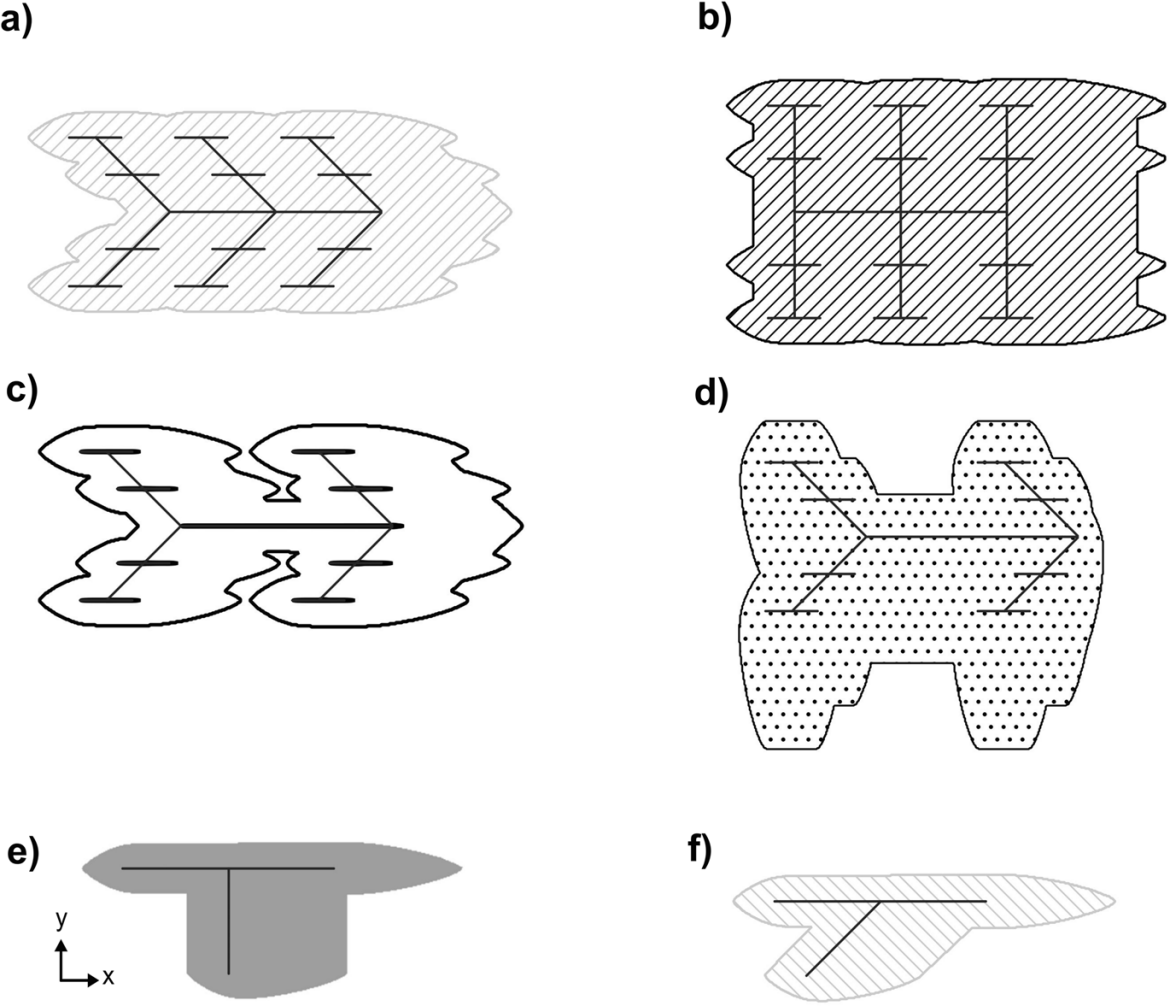
Influence of changing groundwater flow direction (GWD) and intersection angle of secondary pipes to the main pipe (*α*) using a constant leakage of 1 × 10^−7^ m^3^ s^−1^ per m. As a simplification, the edge of the plume represents the total area under risk of pollution. Scenario settings for fractal and synthetic HLSCs (figure parts **a** to **f**) as follows: **a** HLSC with LC III: 1 main pipe, 6 secondary pipes, 24 tertiary pipes. Secondary pipes connected to the main pipe in positions 1, 2, and 3. GWD 1, *α* = 45°. **b** Like **a**, but with *α* = 90°. **c** HLSC LC III: 1 main pipe, 4 secondary pipes, 16 tertiary pipes. Secondary pipes connected to the main pipe in positions 1 and 3. GWD 1, *α* = 45°. **d** Like **c** but with GWD 2. **e** HLSC with LC II: 1 main pipe, 1 secondary pipe. Secondary pipe connected to the main pipe in position 2. GWD 1, *α* = 90°. **f** Like **e**, but with *α* = 45°. GWD 1: along *x*-axis. GWD 2: along *y*-axis

**Fig. 5:**
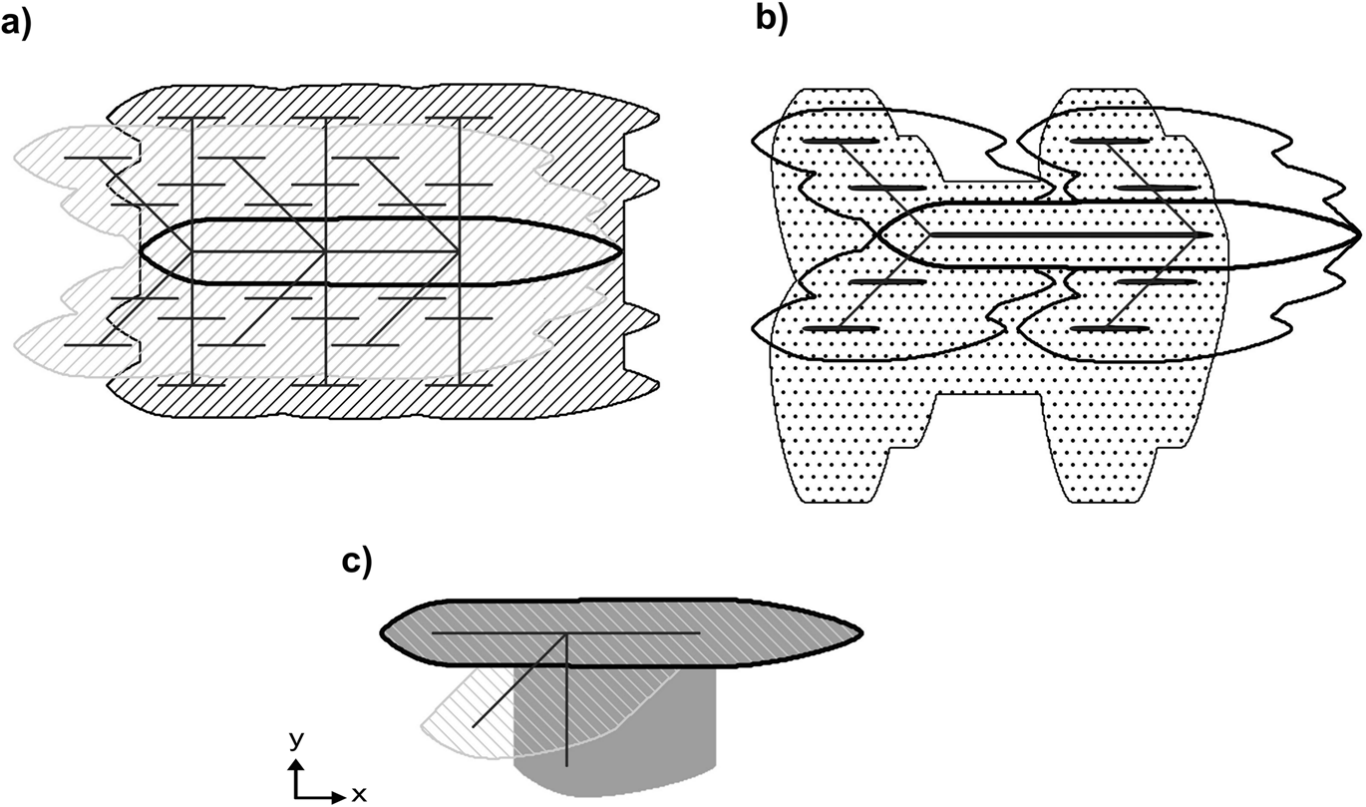
Interception of scenario results obtained from separate HLSC scenarios. Here, three examples related to Fig. 4: **a** interception of results from Fig. 4 a and b; **b** interception of results from Fig. 4 c and d; **c** interception of results from Fig. 4 e and f. A comparison to the base case scenario is shown as well

When compared to the area of the plume generated by the BCS (i.e., *A*_*p*, BCS_), adding new HLSCs following fractal geometries (LCs II and III) increases RA_*j*_ values by 60 to 690%. The shape of the plume greatly varies depending on HLSC’s characteristics (selected examples are shown in Fig. 4, as well as their overlapping in Fig. 5). The intersection angle (*α*), the connection position of the secondary pipe to the main pipe (PS), the level of HLSC complexity (LC), the amount of secondary or tertiary pipes (*n*_pipes_), and the total length (*L*) show recognizable influence on contaminant spreading. When secondary pipes are intersected with the main pipe at 90°, scenarios with different LC values showed that the resulting area of the plume is larger than for the same configuration at 45° (Fig. 4 a, b and e, f). Hence, varying *α* in a given set of HLSCs increases *A*_*p*, SC_ by between 30 and 230%. Additionally, the shape of the contaminant plume changes, depending on the location of the HLSC relative to the groundwater flow direction (GWD). HLSCs positioned perpendicular to GWD generate a larger spreading of contaminants towards the flow gradient. For instance, given the same HLSC, changing GWD can increase the area of the plume by up to 42% (compare Fig. 4 c and d; see Table S6). The center of the contaminant front for each cell moves with the average groundwater flow direction, creating an enlarged contaminant plume. Hydrodynamic dispersion generates a spreading of the plume transversal to the GWD for each HLSC. In this study, the contaminant plume evolution of conservative contaminants was analyzed using the area of the generated plume as reference. As expected, since advection dominates the conservative contaminant transport in porous media (Fetter, 1993), the spreading of contaminants was highly influenced by the direction of the groundwater velocity vectors. Considering that sewer-borne contaminants are transported mainly by advection, remediation efforts should consider not only addressing the source of exfiltration (i.e., specific leakage points within a pipe) but also managing the potential migration pathways of contaminants in the urban aquifer. In the examples shown in Fig. 4, intensive monitoring and pipe inspection efforts may be allocated in those sections of sewer networks located perpendicular to the groundwater flow gradient due to the large spreading of contaminants.

#### Significant factors affecting contaminant spreading in the aquifer

**Fig. 6:**
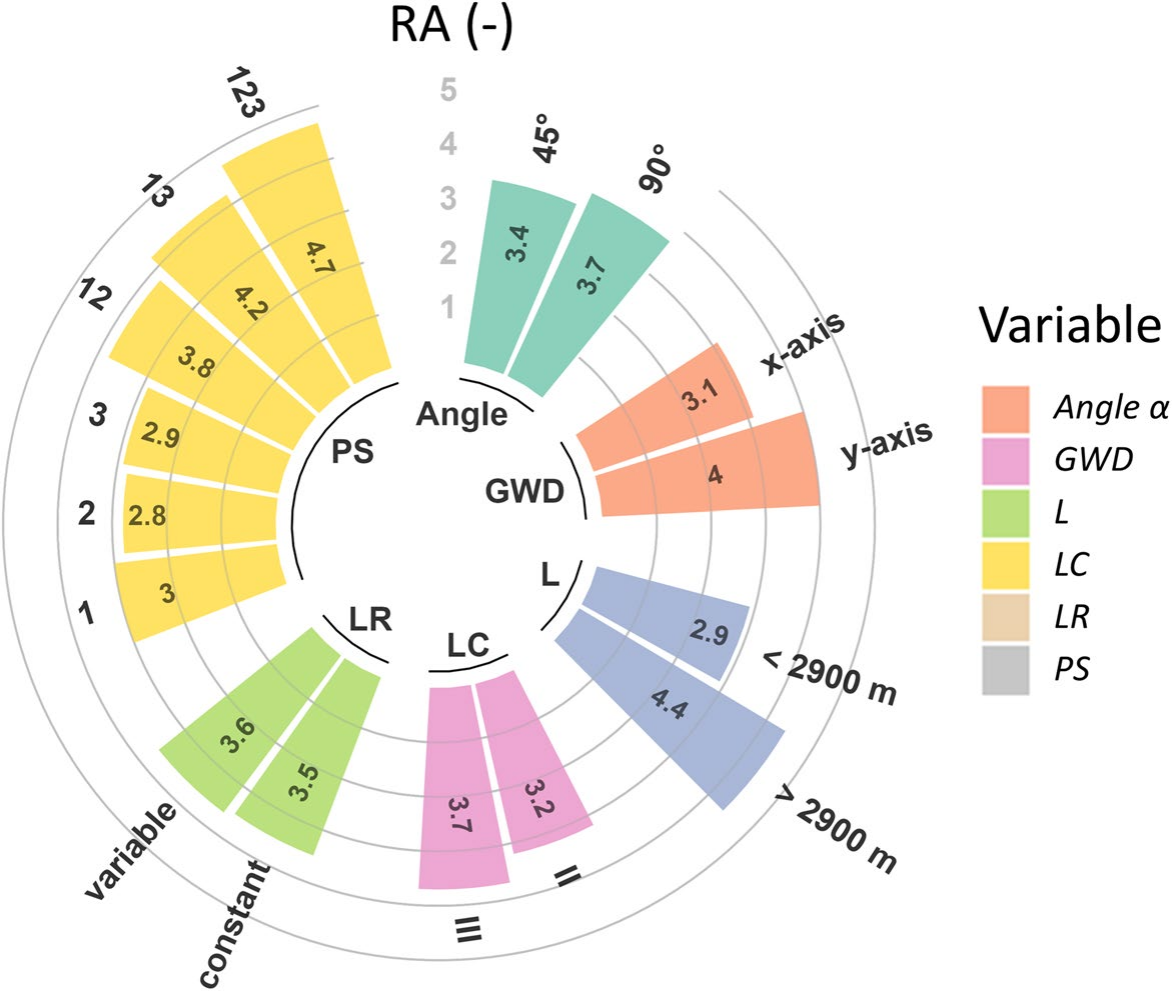
Arithmetic mean values of the relative areas RA_*j*_ (-) of contaminant plume generated by each scenario. Intersection angle (*α*) (°): angle of connection to the main pipe; GWD 1: groundwater direction along *x*-axis; GWD 2: groundwater direction along *y*-axis; *L* (m): total length of HLSC; LC: level of complexity of fractal UDN; LR1: constant leakage rate; LR2: variable leakage rate; PS: position of connection of the secondary pipe to the main pipe; PS 123: secondary pipes connected to positions 1, 2, and 3; PS 13: secondary pipes connected to positions 1 and 3; PS 12: secondary pipes connected to positions 1 and 2; PS 1, PS 2, and PS 3: secondary pipe connected to positions 1, 2, and 3, respectively. Number of scenarios excluding the variations of the BCS: 284

While evaluating the mean values of the relative area of the contaminant plume (i.e., RA_*j*_; Eq. 2) obtained for each scenario group, results suggested that, among the HLSC characteristics, *L* has the highest influence on plume response, with a difference in arithmetic means of 52% (Fig. 6). Among all HLSCs evaluated, those covering the largest drainage area (*A*_HLSC_) (e.g., secondary pipes connected in position *PS 123*), having the majority of secondary pipes intersected at 90° and a length larger than 2900 m, have the relatively largest contaminant plumes. Hence, those geometric characteristics of HLSCs can be used as additional indicators to prioritize UDNs for condition assessment using a risk-based approach (Tscheikner-Gratl et al., 2019). It was observed that the physical arrangement, i.e., the layout, of the sewer network has an important role in the shape of the contaminant plume. Therefore, it is expected that highly branched UDN topologies are related to elongated contaminant plumes (see Reyes-Silva et al. (2020, 2021) for real examples of UDN topologies). However, further evaluations are needed to analyze the influence of the number of pipes connected to junctions in a UDN (also known as “meshness” or connectivity; Reyes-Silva et al., 2020).

**Fig. 7:**
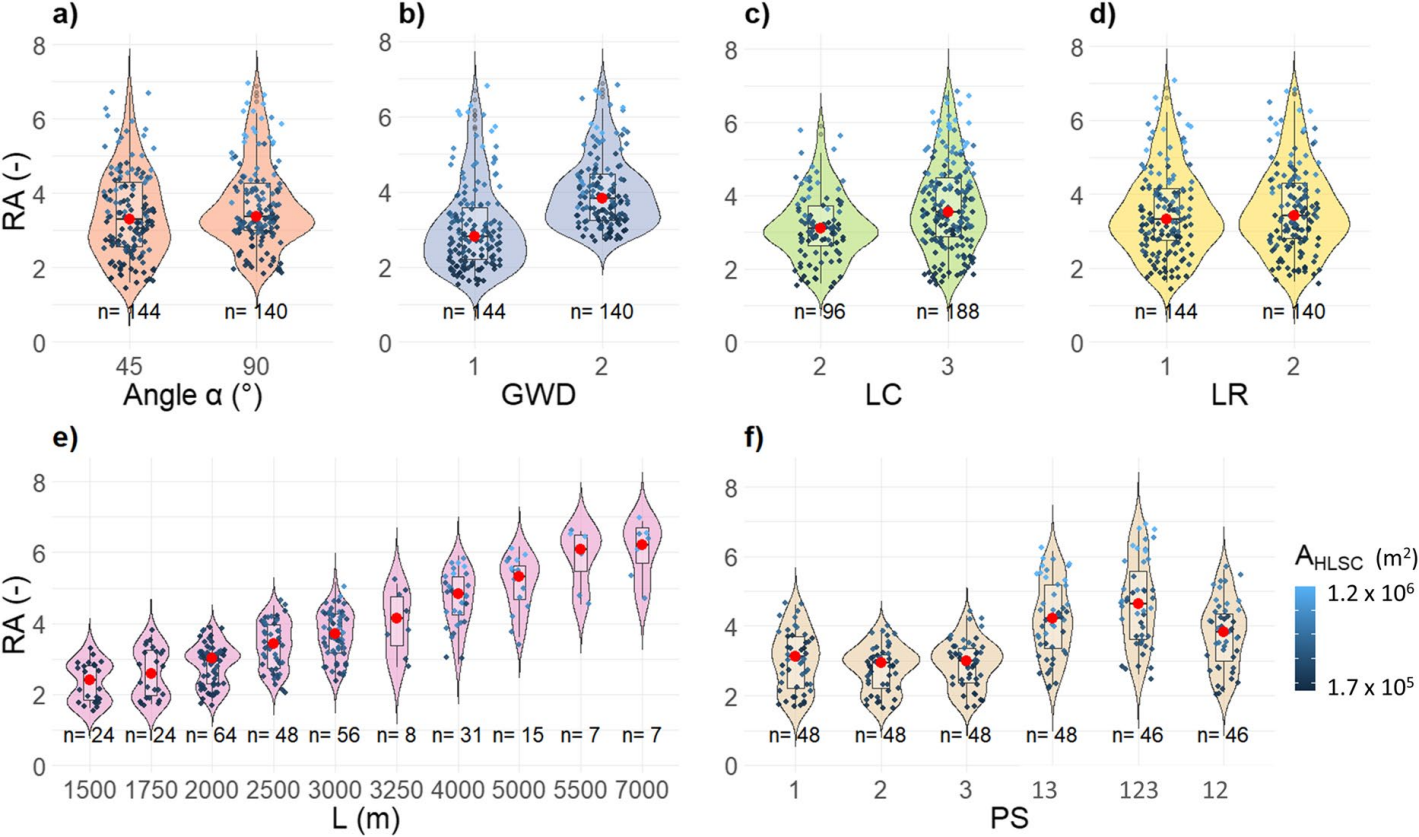
Distribution of relative areas of contaminant plumes RA_*j*_ (-) generated by each scenario. The density curve of the violin plot shows the approximated frequency of data points. Red dot shows the median value of a given group. The color saturation of the jitter points shows decreasing drainage area (*A*_HLSC_) of the evaluated HLSC. Points with brighter color represent larger *A*_HLSC_. **a** Intersection angle *α* of 45° and 90°; **b** GWD 1: groundwater direction along *x*-axis, GWD 2: groundwater direction along *y*-axis; **c** LC: level of complexity of HLSC; **d** LR1: constant leakage rate, LR2: variable leakage rate; **e** *L* (m): total length of HLSC; **f** PS: position of connection of the secondary pipe to the main pipe, i.e., PS 1, PS 2, and PS 3: secondary pipe connected to positions 1, 2, and 3, respectively; PS 13: secondary pipes connected to positions 1 and 3; PS 123: secondary pipes connected to positions 1, 2, and 3; PS 12: secondary pipes connected to positions 1 and 2

The ANOVA showed five factors explaining 89% of the variance of RA_*j*_ with a confidence level of 95% (Table S7). Those factors are level of complexity (LC), total length of HLSC (*L*), intersection angle with a secondary pipe (*α*), position of connection of the secondary pipe to the main pipe (PS), and groundwater flow direction (GWD). The ANOVA demonstrates that these factors have a significant effect on the total area of the plume (*p* value < 0.05). Additionally, *A*_HLSC_ and RA_*j*_ showed a Pearson correlation coefficient of 0.81 (see Table S8). This means that HLSCs covering a larger drainage area generate larger spreading of contaminants (see jitter points with a brighter blue in Fig. 7). This also applies for longer HLSCs (*L* > 2900 m).

This might have two main implications in terms of sewer leakage management and urban groundwater monitoring. First, the prioritization of sewer network assessment areas can be further developed from a condition to a risk-based approach (Tscheikner-Gratl et al., 2019). Consequently, basic characteristics of the layout of a UDN (i.e. *L*, angle α and *A*_HLSC_) can be used as an initial indicator for prioritizing and selecting sections for pipe condition assessment, considering the extend of the contaminant plumes that can be generated if failures occur.

Second, in terms of groundwater monitoring and analysis of plume development due to sewer exfiltration, the HLSC can be characterized by focusing solely on the UDN’s total length (*L*), which is related to the number of pipes connected within the UDN and drainage area (*A*_HLSC_). Hence, knowledge about both the HLSC characteristics and the groundwater flow direction is important to predict *A*_*p*, SC_. This will be exemplified in the next section.

In summary, GWD has a dominant influence on the spreading of the contaminants (*p* value < 0.05; Table S9), since advection primarily controls the conservative contaminant transport (Fig. 7b; Fetter, 1993). In contrast, LR variations have minimal effects on the shape of the long-term contaminant plume (*p* value > 0.05; Table S9, Fig. 7d), showing that it remains relatively consistent regardless of the specific LR value. Accordingly, to delineate contaminant plumes, it may not be necessary to calculate exact exfiltration flow rates. This applies during continuous exfiltration and conservative transport, disregarding the concentration distribution inside the plumes. When comparing two leakage scenarios having identical HLSCs, the mass fluxes in the aquifer consequently differ, since variation of the exfiltration rate will lead to a change of the injected mass (see Table S10). However, the normalized shape of the contaminant plume remains the same and values for RA_*j*_ do not indicate a significant change.

### Application of the parsimonious approach to simulate sewer exfiltration as HLSC: using a real-world UDN layout to analyze contaminant spreading

**Fig. 8:**
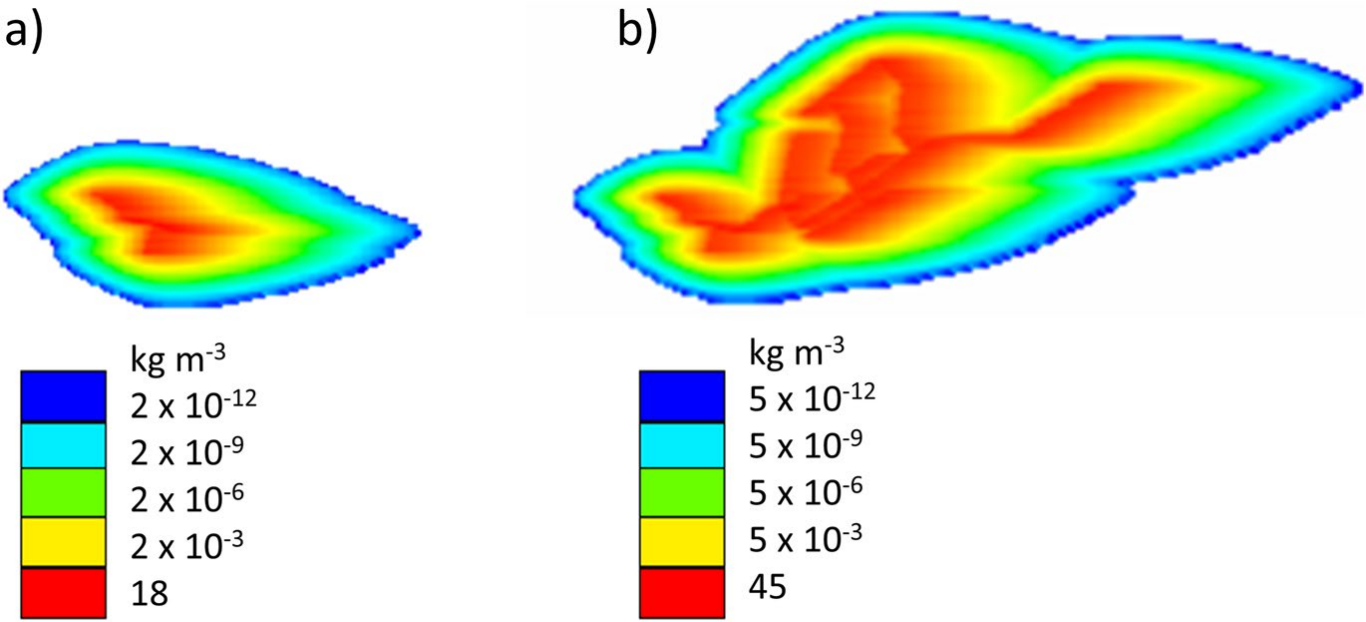
Distribution of the contaminant concentration for two types of HLSC simulated based on an actual UDN part in Dresden, Germany: **a** plume generated by the shorter HLSC (type 5, *L =* 330 m); **b** plume generated by the whole HLSC (type 7, *L =* 2910 m)

The proposed parsimonious approach to simulate sewer exfiltration as HLSC was applied to delineate contaminant plumes, generated by HLSC, following a real-world UDN layout. Hence, this section shows an example on how an actual UDN can be simulated as HLSC. Given the same GWD, simulations of each part of the sewer network showed different plume shapes (Fig. 8). As expected and similar to our findings regarding the synthetic scenarios (see previous section), all plumes expand along the GWD. Additionally, the plume generated by the whole leaky UDN (HLSC type 7) comprises all the plumes associated to each individual HLSC. After relating the areas of the plumes generated by each HLSC to the whole leaky UDN, it was demonstrated that long HLSCs covering a larger drainage area *A*_HLSC_ are associated with larger contaminant plumes, as shown before for the synthetic scenarios. In this example, the largest contaminant plume is generated by the HLSC type 1 (*L=* 890 m), which covers 62% of the total area *A*_HLSC_ under risk of pollution. From a practical point of view and considering a risk-based approach, this means that the HLSC type 1 (subnetwork 1 in Fig. 3) may be prioritized for further pipe condition assessment or higher maintenance efforts. The exfiltration of contaminants originating from this individual HLSCs may create the largest contaminant plume. Hence, RA_*j*_ can be added as a criterion for the development of a vulnerability hotspot mapping of sewer exfiltration and aquifer contamination. Characteristics of the contaminant plume and their relation to the plume generated by the whole leaky UDN are shown in Table S3 and Figure S3. Furthermore, the injected mass after 10 years of simulation varied between 1313 and 3932 tons. The minimum injected mass is associated to the shortest HLSC (type 5, *L =* 330 m), whereas the maximum injected mass was reached when the entire network is simulated (type 7, *L =* 2910 m). Hence, the distribution of the solute concentration within the plume is influenced by the length of HLSC. After simulating the whole UDN (HLSC type 7), the accumulated concentration inside the edge of the plume varies between 2 × 10^−12^ and 45 kg m^−3^ (Fig. 8). By focusing solely on the shape of the plume and disregarding its concentration, it is observed that the plume’s shape remains identical while its area expands with increasing concentration (see Figure S4). This suggests that the geometry of the plume can serve as an initial indicator for identifying areas at risk for contamination and related them to HLSCs. In large urban systems, the process of modeling sewer exfiltration and its associated impacts on groundwater is still limited and not fully understood (Nguyen et al., 2021). Our study shows how a parsimonious approach to simulate sewer exfiltration, representing leaky UDNs as HLSCs, can help in obtaining further information about the potential spreading of contaminants in urban areas, under minimum data requirements. The later strategy can serve as a tool for supporting management of both sewer condition assessment and groundwater monitoring. Understanding that the UDN’s layout strongly influences the shape of the plume may reduce uncertainties related to prioritizing areas for early sewer inspection and developing further vulnerability hotspot mapping. Thus, modeling results may better represent large spatial patterns than point observations taken by aquifer monitoring wells at the city scale.

### Further implications for urban groundwater monitoring and sewer leakage management

#### Delineating potentially polluted regions in urban aquifers considering a real-world UDN layout

**Fig. 9:**
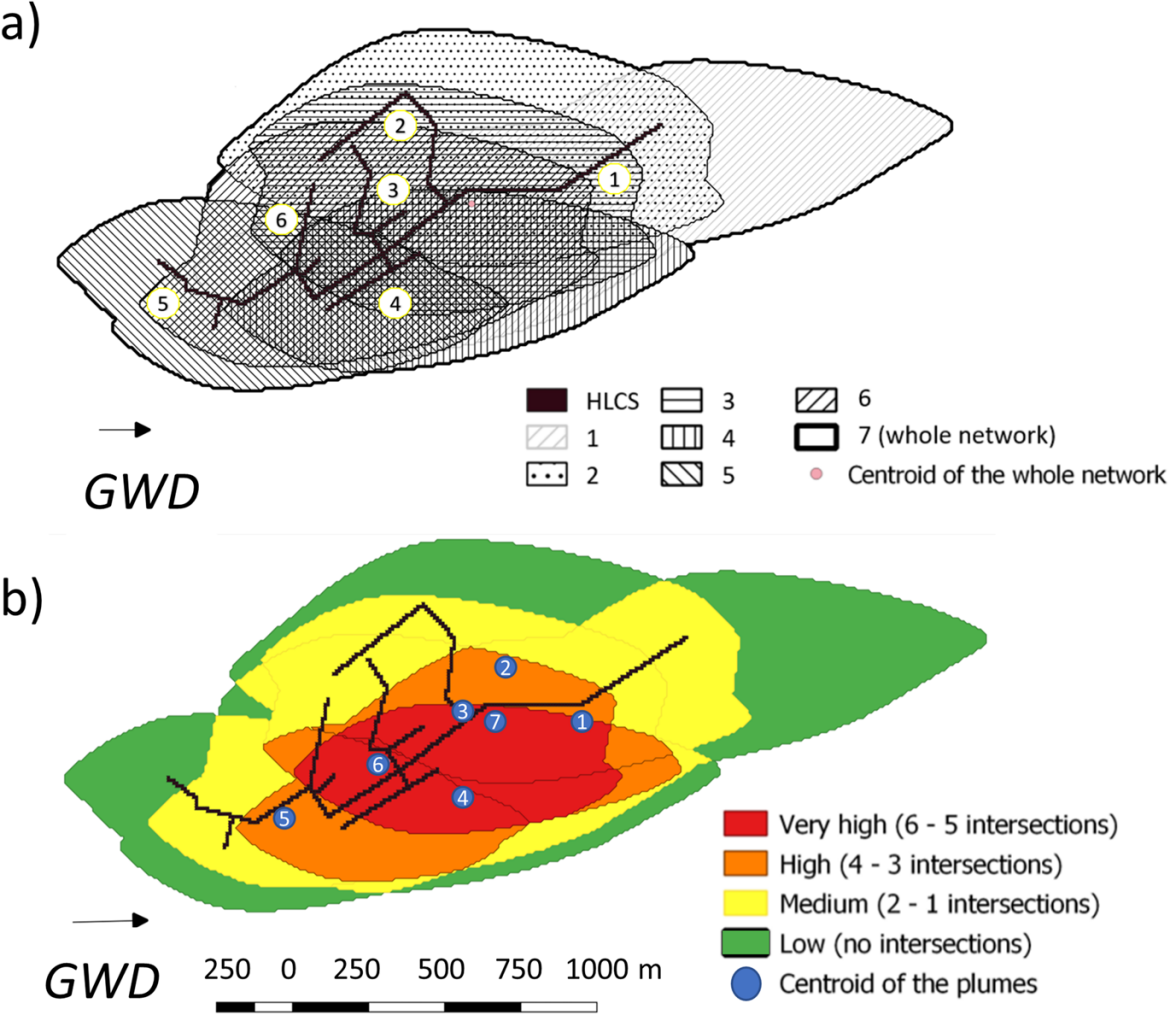
Areas with high risk for pollution due to the intersection of contaminant plumes. **a** Edges and covered areas of the contamination plumes generated by each subnetworks 1 to 6 as well as from the whole network (subnetwork 7). **b** Definition of overlapping areas by intersection of the plumes from subnetworks 1 to 6, showing the geometrical centroids of generated contamination plumes

Using the HLSC approach to simulate sewer exfiltration can facilitate delineating risk areas in urban aquifers. Considering the influence of HLSCs on contaminant spreading, the real-world UDN layout was used to exemplarily identify areas of potential risk for contamination and, based on that, to recommend point-based monitoring locations (typically given by observation wells). The distance separating each HLSC from another influences the overlap of the contaminant plumes. Hence, HLSCs adjacent to each other generate a larger intersected area. For instance, since the HLSC types 2–4 and 6 are located nearby (maximum separation of 300 m), their contaminant plumes overlap each other. Additionally, all HLSC are connected to HLSC type 1 (main pipe). Hence, its contaminant plume intersects with all the others. We identified and classified four groups of areas under pollution risk, considering the number of intersected plumes: low risk (no intersections or no plumes at all), medium risk (1 to 2 intersected plumes), high risk (3 to 4 intersected plumes), and highest risk (5 to 6 intersected plumes). The plume of HLSC type 7 (entire UDN) was not included in the analysis, since its area comprises all other contaminant plumes. This simplification for defining areas at risk of pollution assumes that when the plumes mix (potentially with different constituents, concentrations, and chemical milieus), synergistic or additive effects can occur increasing the potential hazard. Those effects can even lead to either inhibit or enhance degradation processes. However, it neglects a possible generation of decay products that could be even more hazardous than the original components. The region with “highest risk” for pollution, colored red in Fig. 9, represents around 39% of the total contaminant area. This region expands towards the flow gradient. This is especially important, since monitoring studies at city scale (e.g., Rusiniak et al., 2021) have already reported the relative positioning of the observation wells and leaky sewer pipes with respect to the direction of groundwater flow as key information needed for effective monitoring. To ensure effective monitoring, only observation wells situated along the groundwater flow direction can be utilized to detect sewage leakages and the presence of emerging contaminants (Rusiniak et al., 2021).

All geometric centroids of the contaminant plumes generated by the individual HLSC are located in areas with *high* to *highest risk* for pollution (Fig. 9b). This is useful from a practical point of view, since the coordinates of the geometric centroids can be used as a first reference for selecting possible observation points for long-term groundwater quality monitoring. This would facilitate the identification of emerging contaminants, within the aquifer, associated to leaky UDN, because at those points the probability to detect the contaminants may be higher. However, this should be further studied. As a simplification, in Fig. 9b, the geometrical centroid of the plume generated by the whole UDN layout (subnetwork 7) may be the first potential position of a monitoring well. This point is located within the *highest risk* region of pollution and was obtained by simulating the exfiltration from the whole UDN. Using this parsimonious approach to identify regions of *risk of pollution*, we could overcome one of the main challenges of groundwater monitoring: selecting observation points covering representative areas for monitoring (Rusiniak et al., 2021; Stigter et al., 2011). However, the geometric centroids of the simulated contaminant plumes are not the same as the mass centers of the actual contaminant plumes. Therefore, further evaluations to optimize groundwater quality monitoring points should be done. These evaluations should consider that the positioning of groundwater observation points for a given UDN layout can be constrained by the existing urban infrastructure and strongly depending on land use, accessibility, and property owner rights.

Other methods used to predict polluted areas within aquifers due to leaky UDN were focused on the structural conditions of sewer networks and exfiltration probabilities of pipes (Lee et al., 2015; Roehrdanz et al., 2017). However, these approaches require a detailed description of the UDN’s structural conditions and aquifers beneath these networks. Hence, their application is difficult at city scale. In contrast, our parsimonious method facilitates the identification of regions with high pollution risk for a given UDN, without prior knowledge of leakage locations (point sources for pollution), hydraulic leakage rates, or structural conditions of the sewer network. It requires analyzing the HLSC’s shape (e.g., location and length) and groundwater flow direction. This parsimonious approach, i.e., (i) focusing on plume’s geometry rather than concentration distributions; and (ii) skipping the percolation process through the vadose zone, may overestimate the size of areas effectively affected by pollution. However, it leads to a more straightforward way to identify possible locations of monitoring wells at city scale, which is especially important from a practitioner’s point of view. Hence, this approach may be useful for recommending preliminary areas for potential groundwater sampling points in places where no urban monitoring systems exist and access to information is limited due to budgetary constraints.

#### Sewer assessment: from condition to a risk-based approach under minimum data requirements

As previously discussed, structural conditions of sewer networks and exfiltration probabilities of pipes are rarely available at city scale (Nguyen et al., 2021). Our parsimonious approach to simulate sewer exfiltration as HLSC can be used to analyze the spatiotemporal distributions of water-dissolved, sewer-borne contaminants. For real and synthetic UDN layouts presented in previous sections, it was observed that HLSCs positioned perpendicular to GWD generate a larger spreading of contaminants towards the flow gradient. Additionally, geometric characteristics of the layout of a UDN (i.e. LC, *L*, angle *α*, and *A*_HLSC_) significantly affect the generated contaminant plume. Hence, considering a risk-based approach for sewer condition assessment (Tscheikner-Gratl et al., 2019), it can be important to analyze the characteristics of the UDN layout and their relationship to the GWD for prioritizing strategic areas for sewer network assessment. It is expected that, in the long term, highly branched UDNs (i.e., with long UDN length *L*) generate a broader spread of contaminants (*A*_*p*_). Therefore, when scarce information is available, we recommend to target maintenance and structural health inspection efforts to those sections of sewer networks covering higher drainage areas and having the longest pipe lines perpendicular to the groundwater gradient. This can be a first step for further development of vulnerability maps for leakage management at city scale (Sadeghikhah et al., 2022).

Considering our results for homogeneous aquifers and assuming that the local hydrogeology conditions do not influence the plume evolution, a tool to automatically predict polluted regions in urban aquifers using open-access data can be generated. This can include the automatize generation of more realistic UDN layouts using Open Street Map (Reyes-Silva et al., 2023) and generation of contaminant plumes via GIS. It can be implemented by solely considering the UDN’s geometry and groundwater flow direction, instead of performing groundwater contaminant transport simulations. Stakeholders can use this tool for preliminary studies to analyze plume evolution. This can be valuable in urban areas with limited data availability, facilitating the prioritization of strategic interventions for sewer network assessments. We suggest to create a proof of concept using a real-world case study for sewer condition assessment from a risk-based perspective.

## Conclusions

Understanding spatiotemporal distributions of sewer-borne contaminants in the subsurface is essential for the development of efficient point-based monitoring strategies. This, in turn, allows protecting urban aquifers and preventing detrimental effects on both humans and ecosystems. In this study, we evaluated the relation between UDN layouts, represented as HSLCs, and areas potentially affected by contaminant plumes in aquifers. This was done by characterizing the shape of contaminant plumes using long-term contaminant transport modeling and geospatial analyses. The scenarios considered described fractal (synthetic) and actual UDN layouts with variations in leakage rate and groundwater flow direction. We demonstrated a significant influence of the UDN geometry on plume response. Since advection primarily drives the spreading of contaminants during conservative transport, our statistical analysis demonstrates the importance of describing the position of the HLSC relative to the predominant groundwater flow direction. Under near-homogeneous and isotropic hydrogeological characteristics, the length and distribution of pollution sources (here the leaky UDNs represented as HLSCs) are the main factors influencing the spreading of sewer-borne contaminants. In contrast, the influence of the distribution of leakage rates in the network is not statistically significant (for the scenarios investigated), having a minimal effect on the shape and total area of the contaminant plume.

Identifying that UDN’s layout has a significant influence on plume response is crucial for accurately assessing and managing urban groundwater contamination risks. Considering this, a real-world UDN layout, represented by multiple intersected HLSCs, was introduced to apply the parsimonious approach to predict polluted areas in urban aquifers. The implemented approach relied on the UDN’s layout and selected basic hydrogeological information only (e.g., groundwater flow direction). Hence, it reduced the requirement for comprehensive data traditionally needed to represent interactions between sewers and groundwater at urban scales. The approach presented can facilitate the identification and localization of sewer-borne contaminant plumes at scales of city districts and cities. It can, therefore, be used in future studies to recommend preliminary positioning of observation wells for groundwater monitoring or to support sewer assessment management by switching from a condition- to a risk-based approach. Although realized via a numerical model in this study, using particle tracking methods, as implemented in many GIS systems, would also be feasible for analyzing plume evolution. One of the disadvantages of our parsimonious approach is given by a potential overestimation of polluted areas. Hence, this method should be validated considering a real-world case study incorporating information about hydrogeological conditions, groundwater-based measurements of contaminant concentration, and sewer network layout. This should include analyzing the influence of physical heterogeneities of the aquifer on the contaminant plume evolution.

### Supplementary information

ESM 1 (PDF 815 kb)

## Data Availability

The datasets generated and/or analyzed during the current study are available from the corresponding author on reasonable request.

## Declarations

All authors have read, have understood, and have complied as applicable with the statement on “Ethical responsibilities of Authors” as found in the Instructions for Authors.
