# Supplementary material for: A parsimonious approach to predict regions affected by sewer-borne contaminants in urban aquifers
Source: Environ Monit Assess. 2023 Nov 23;195(12):1517. doi: 10.1007/s10661-023-12027-6 (PMC10665238; doi:10.1007/s10661-023-12027-6)
Supplement: Supplementary file 1 — (PDF 815 kb) [file 10661_2023_12027_MOESM1_ESM.pdf]

Journal: Environmental Monitoring and Assessment

Predicting Polluted Regions in Urban Aquifers: A Parsimonious Approach to Simulate Sewer Exfiltration

Karen L. Rojas-Gómez [1, 2, *], Martin Binder [3, 4], Marc Walther [5], Christian Engelmann [3]

[1] Helmholtz-Centre for Environmental Research – UFZ, Department of Aquatic Ecosystem Analysis, Brückstraße 3a, 39114 Magdeburg, Germany
[2] Technische Universität Dresden, Institute of Urban Water Management, Bergstraße 66, 01069 Dresden, Germany
[3] Technische Universität Bergakademie Freiberg, Institute of Geology, Chair of Hydrogeology and Hydrochemistry, Gustav-Zeuner-Straße 12, 09599 Freiberg, Germany
[4] University of Basel, Department of Environmental Sciences, Research Group of Applied and Environmental Geology, Bernoullistrasse 32, 4056 Basel
[5] Technische Universität Dresden, Institute of Forest Growth and Forest Computer Sciences, Pienner Straße 8, 01737 Tharandt, Germany

***** Correspondence: karen.rojas@ufz.de

ORCID: K.L. R.-G.: 0000-0001-7097-7595; M. B.: 0000-0003-2296-0582; M. W.: 0000-0002-3180-1551; C. E.: 0000-0002-7948-3484

Electronic Supplementary Material

This document provides additional information of methods and results, including figures and tables.

Table supplements [Table S1 to S10]

Table S1. Characteristics of the HLSC representing one single pipe. Level of complexity - *LC* 1. All HLSC of length 1000m have the same geometric centroid and different angle formed by the HLSC and the flow vectors or width. When the width of the HLSC changes, the HLSC represents a new area of leakage

HLSC	Angles of intersections (α) (°)	Width (m)	Centroid *x*	Centroid *y*
1	180	5	1870	1627
2	90	5	1870	1627
3	45	5	1870	1627
4	135	5	1870	1627
5	180	250	1870	1627
6	180	500	1870	1627

Table S2. HLSC characteristics of artificial sewer network. Levels of complexity -*LC* 2 and 3. Depending on the position of the connection of secondary pipe (*PS*) to the main pipe and the number of tertiary pipes, the covered drainage area (A_{HLSC}) and location of the centroid may change

Number of HLSC Layout	Level of complexity *LC*	Angle α (°)	PS	Number of pipes: Main pipe	Secondary pipes	Tertiary pipes	Total pipes	Geometric characteristics: L (m)	Drainage area A_{HLSC} (m^2)	x centroid	y centroid
1	2	45	1	1	1	0	2	1500	174125	1588	1511
2	2	45	12	1	2	0	3	2000	261625	1604	1472
3	2	45	123	1	3	0	4	2500	349988	1697	1452
4	2	45	13	1	2	0	3	2000	349988	1697	1452
5	2	45	2	1	1	0	2	1500	174125	1754	1511
6	2	45	3	1	1	0	2	1500	174988	1922	1511
7	2	45	1	1	2	0	3	2000	470750	1471	1628
8	2	45	12	1	4	0	5	3000	645750	1516	1628
9	2	45	123	1	6	0	7	4000	822475	1614	1628
10	2	45	13	1	4	0	5	3000	822475	1614	1628
11	2	45	2	1	2	0	3	2000	348250	1754	1628
12	2	45	3	1	2	0	3	2000	349975	1922	1628
13	2	90	1	1	1	0	2	1500	246263	1704	1463
14	2	90	12	1	2	0	3	2000	370013	1760	1407

15	2	90	123	1	3	0	4	2500	495000	1873	1380
16	2	90	13	1	2	0	3	2000	495000	1873	1380
17	2	90	2	1	1	0	2	1500	246263	1871	1463
18	2	90	3	1	1	0	2	1500	247500	2039	1463
19	2	90	1	1	2	0	3	2000	495013	1704	1629
20	2	90	12	1	4	0	5	3000	743763	1760	1630
21	2	90	123	1	6	0	7	4000	995000	1873	1630
22	2	90	13	1	4	0	5	3000	995000	1873	1630
23	2	90	2	1	2	0	3	2000	495013	1871	1629
24	2	90	3	1	2	0	3	2000	497500	2039	1629
25	3	45	1	1	1	2	4	1750	217000	1531	1488
26	3	45	12	1	2	4	7	2500	304500	1584	1461
27	3	45	123	1	3	6	10	3250	392563	1689	1446
28	3	45	13	1	2	4	7	2500	392563	1689	1446
29	3	45	2	1	1	2	4	1750	217000	1730	1488
30	3	45	3	1	1	2	4	1750	217563	1931	1488
31	3	45	1	1	2	4	7	2500	600250	1399	1628
32	3	45	12	1	4	8	13	4000	775250	1471	1628
33	3	45	123	1	6	12	19	5500	951375	1579	1628
34	3	45	13	1	4	8	13	4000	951375	1579	1628
35	3	45	2	1	2	4	7	2500	434000	1730	1628
36	3	45	3	1	2	4	7	2500	435125	1931	1628
37	3	45	1	1	1	4	6	2000	227938	1511	1486
38	3	45	12	1	2	8	11	3000	315438	1568	1461
39	3	45	123	1	3	12	16	4000	414000	1689	1446
40	3	45	13	1	2	8	11	3000	414000	1689	1446
41	3	45	2	1	1	4	6	2000	217000	1730	1488
42	3	45	3	1	1	4	6	2000	228063	1947	1486
43	3	45	1	1	2	8	11	3000	600250	1399	1628
44	3	45	12	1	4	16	21	5000	775250	1471	1628
45	3	45	123	1	6	24	31	7000	972375	1594	1628
46	3	45	13	1	4	16	21	5000	972375	1594	1628
47	3	45	2	1	2	8	11	3000	434000	1730	1628
48	3	45	3	1	2	8	11	3000	456125	1947	1628
49	3	90	1	1	1	2	4	1750	310000	1670	1428
50	3	90	12	1	2	4	7	2500	435000	1751	1389
51	3	90	123	1	3	6	10	3250	561250	1871	1368
52	3	90	13	1	2	4	7	2500	561250	1871	1368
53	3	90	2	1	1	2	4	1750	310000	1870	1428
54	3	90	3	1	1	2	4	1750	311250	2071	1428
55	3	90	1	1	2	4	7	2500	682500	1635	1628
56	3	90	12	1	4	8	13	4000	932500	1720	1628
57	3	90	123	1	6	12	19	5500	1245000	1870	1628
58	3	90	13	1	4	8	13	4000	1245000	1870	1628
59	3	90	2	1	2	4	7	2500	620000	1870	1628
60	3	90	3	1	2	4	7	2500	682500	2105	1628
61	3	90	1	1	1	4	6	2000	325625	1652	1425
62	3	90	12	1	2	8	11	3000	450625	1735	1389

63	3	90	123	1	3	12	16	4000	591875	1871	1369
64	3	90	13	1	2	8	11	3000	591875	1871	1369
65	3	90	2	1	1	4	6	2000	310000	1870	1428
66	3	90	3	1	1	4	6	2000	326250	2089	1426
67	3	90	1	1	2	8	11	3000	682500	1635	1628
68	3	90	12	1	4	16	21	5000	932500	1720	1628
69	3	90	123	1	6	24	31	7000	1245000	1870	1628
70	3	90	13	1	4	16	21	5000	1245000	1870	1628
71	3	90	2	1	2	8	11	3000	620000	1870	1628
72	3	90	3	1	2	8	11	3000	682500	2105	1628

Table S3. Characteristics of HLSC derived from real-world UDN (subnetworks 1 to 6 and whole network – type 7) and comparison among generated contaminant plumes. L: Length of the HLSC; A_{HLSC}: Area covered by HLSC; *M*: total injected mass after 10 years of simulation; percentage of injected mass (%); $A_{p,SC}$: area of the contaminant plume; percentage (%) of the area of the plume generated by individual HLSC (type 1 to 6) compared to plume generated by the whole network (type 7); location of centroid of plume (*x* and *y* coordinates)

Characteristics of HLSC					Characteristics of the contaminant plume			
**Type HLSC**	**L (m)**	**A_{HLSC} (m^2)**	**M (kg)**	**Injected mass (%)**	**$A_{p,SC}$ (m^2)**	**(%)**	**Coordinates centroid of plume x_p**	**y_p**
1	890	42030	1312588	33	396863	62	1602	1794
2	520	22873	740276	19	286988	45	1467	1902
3	410	11674	534990	14	246475	39	1395	1809
4	340	6624	416794	11	194163	30	1398	1664
5	330	4144	391911	10	194063	30	1070	1635
6	420	11667	534990	14	272288	43	1444	1802
7	2910	179860	3931584	100	637238	100	1258	1726

Table S4. Characterization of contaminant plumes generated by a single HLSC, level of complexity LC 1, forming four different intersection angles α with the groundwater velocity vectors. The individual plume characteristics are $A_{p,SC}$: area of the contaminant plume, location of centroid of plume (x and y coordinates), width and length of the plume (wp and Lp respectively); RA_j represents the relative areas of contaminant plumes

**HLSC Characteristics**				**Plume characteristics**					**Comparing plumes to base case scenario**
**Angle α (°)**	**Width w_{SC} (m)**	**Centroid x_{HLSC}**	**Centroid y_{HLSC}**	**$A_{p,SC}$ (m^2)**	**Centroid Plume x**	**Centroid Plume y**	**Width - wp (m)**	**Length - Lp (m)**	**RA_j (-)**
180	*5*	1870	1627	370850	2043	1627	240	1780	1.0
90	5	1870	1627	855025	2044	1630	1230	740	2.3
45	5	1870	1627	641900	2050	1621	1300	736	1.7
135	5	1870	1627	641838	2050	1634	1300	736	1.7

Table S5. Characterization of the contaminant plume generated by one HLSC having different widths and two different intersection angles α with the groundwater velocity vectors. The individual plume characteristics are $A_{p,SC}$: area of the contaminant plume, location of centroid of plume (x and y coordinates), width and length of the plume (wp and Lp respectively); RA_j: represents the relative areas of contaminant plumes

**HLSC Characteristics**					**Plume characteristics**					**Comparing plumes to base case scenario**
**HLSC**	**Angle α (°)**	**Width (m)**	**Centroid x_{HLS}**	**Centroid y_{HLS}**	**$A_{p,SC}$ (m^2)**	**Length (m)**	**Width (m)**	**Centroid x**	**Centroid y**	**RA_j (-)**
1H-A	0	5	1870	1627	370850	1780	240	2043	1627	1.0
1H-B	0	250	1870	1627	807200	1780	490	2043	1627	2.2
1H-C	0	500	1870	1627	1252400	1780	730	2043	1627	3.4
1V-A	90	5	1870	1627	855025	750	1230	2044	1630	2.3
1V-B	90	250	1870	1627	1216838	1030	1240	2044	1630	3.3
1V-C	90	500	1870	1627	1564713	1300	1240	2044	1630	4.2

Table S6. Characteristics of HLSC and generated contaminant plumes shown in Figure 4. LC: Level of complexity of fractal UDN (1, 2 or 3); Intersection angle α: angle of connection to main pipe; *PS*: position of connection of secondary pipe to main pipe, *PS 123*: secondary pipes connected to positions 1, 2 and 3; *PS 13*: secondary pipes connected to positions 1 and 3; *PS 12*: secondary pipes connected to positions 1 and 2; *PS 3/ PS 2/PS 1*: secondary pipe connected to positions 3 / 2 / 1 respectively; L: total length of HLSC; N_{pipes}: number of connected pipes; x_{HLSC}: coordinate x for the centroid of the HLSC representing the sewer network; y_{HLSC}: coordinate y for the centroid of the HLSC representing the sewer network; A_{HLSC}: area covered by the HLSC representing the UDN; GWD 1: groundwater direction along x-axis; GWD 2: groundwater direction along y-axis; *LR1*: constant leakage rate; $A_{p,SC}$: total area of the contaminant plume; *xp*: coordinate x for the centroid of the contaminant plume; *yp*: coordinate y for the centroid of the contaminant plume; RA_j: relative area of the contaminant plume for a given HLSC in relation to the base case scenario

	HLSC characteristics								Other factors		Plume characteristics			Comparison to base case
**Sc**	**LC**	**Angle α (°)**	***PS***	***L* (m)**	**N_{pipes}**	**x_{HLSC}**	**y_{HLSC}**	**A_{HLSC} (m²)**	***GWD***	***LR***	**$A_{p,SC}$ (m²)**	***xp***	***yp***	**RA_j**
BC	1	-	-	1000	1	1870	1627	-	1	1	370850	2043	1627	1
Fig 4a	3	45	123	7000	31	1594	1628	972375	1	1	1732350	1828	1627	4.7
Fig 4b	3	90	123	7000	31	2075	1627	1245000	1	1	2316250	2075	1627	6.2
Fig 4c	3	45	12	5000	21	1471	1628	775250	1	1	1333275	1640	1627	3.6
Fig 4d	3	45	12	5000	21	1471	1628	775250	2	1	1978400	1519	1417	5.3
Fig 4e	2	90	2	1500	2	1871	1463	246263	1	1	706938	2043	1461	1.9
Fig 4f	2	45	2	1500	2	1754	1511	174125	1	1	594650	1940	1524	1.6

Table S7. ANOVA used to identify the factors with a significant influence on the spreading of the contaminants RA_j. Factors: groundwater flow direction (GWD); total length of HLSC (L); Level of complexity of fractal UDN (LC); position of connection of secondary pipe to main pipe (PS); and area covered by the HLSC (A_{HLSC}). The variable response was RA_j. Adjusted R-squared: 0.8903, shows that the model explains 89% of the variability RA_j. The ANOVA

shows a test of statistical significance that compares the mean square versus the estimated experimental error. In this case all factors have a p-value less than 0.05 (see column *Pr(>F)*), showing that their means are significantly different than zero with a 95% of confidence level. By including the GWD, the model explains an additional 18% of the variance, compared to a model without including GWD

Variable	*Df*	*Sum Sq.*	*Mean Sq.*	*F value*	*Pr(>F)*
LC	1	20.947	20.947	141.198	< 2.2e-16
L	1	227.794	227.794	1535.521	< 2.2e-16
α	1	7.42	7.42	50.015	1.25e-11
A_{HLSC}	1	16.167	16.167	108.981	< 2.2e-16
PS	1	1.887	1.887	12.72	0.00043
GWD	1	67.281	67.281	453.529	< 2.2e-16
Residuals	277	41.093	0.148		

Table S8. Pearson correlation matrix among HLSC and contaminant plume characteristics. LC: Level of complexity of fractal UDN (1, 2 or 3). L: total length of HLSC; N_{pipes}: number of connected pipes; A_{HLSC}: area covered by the HLSC representing the UDN; α: angle of connection of secondary pipe to main pipe; RA_j: relative area of the contaminant plume. Length of the HLSC is positive correlated to the number of pipes, area covered by the HLSC and the area of the plume. These linear correlations are high (Pearson correlation coefficient higher than 0.5). Additionally, the area covered by the HLSC has a high linear correlation with the number of connected pipes and area of the plume

	LC	L	N_{pipes}	A_{HLSC}	α	RA_j
LC	1.00	0.35	0.54	0.17	-0.01	0.23
L	0.35	1.00	0.94	0.83	-0.03	0.80
N_{pipes}	0.54	0.94	1.00	0.72	-0.03	0.71
A_{HLSC}	0.17	0.83	0.72	1.00	0.24	0.81
α	-0.01	-0.03	-0.03	0.24	1.00	0.12
RA_j	0.23	0.8	0.71	0.81	0.12	1.00

Table S9. Testing the statistically significant differences between the means of the relative area of the plume (RA_j) obtained by the scenarios, in which the groundwater direction (GWD) and leakage rate (LR) were varied using a t-test. GWD 1: groundwater direction along x-axis; GWD 2: groundwater direction along y-axis; LR 1: constant leakage rate; LR 2: variable leakage rate; p-value<0.05 represents a statistically significance with 95% of confidence level

**Parameter**	**Mean RA_j**	**p value for RA_j**
GWD 1	3.10	5.49×10^{-12}
GWD 2	4.01	
LR 1	3.50	0.4582
LR 2	3.60	

Table S10. Comparing two HLSC having same geometry, groundwater flow direction (GWD) but different leakage rate (LR). BC shows the base case scenario. LC: Level of complexity of fractal UDN (1 or 2); Intersection angle (α): angle of connection to main pipe; PS: position of connection of secondary pipe to main pipe, PS 12: secondary pipes connected to positions 1 and 2; L: total length of HLSC; N_{pipes}: number of connected pipes; x_{HLSC}: coordinate x for the centroid of the HLSC representing the sewer network; y_{HLSC}: coordinate y for the centroid of the HLSC representing the sewer network; A_{HLSC}: area covered by the HLSC representing the UDN; GWD 1: groundwater direction along x-axis; *LR1*: constant leakage rate; *LR2*: variable leakage rate; $A_{p,SC}$: total area of the contaminant plume; *xp*: coordinate x for the centroid of the contaminant plume; *yp*: coordinate y for the centroid of the contaminant plume; RA_j: relative area of the contaminant plume

**Sc**	**HLSC characteristics**								**Other factors**		**Plume characteristics**			**Comparison to base case**	**Max concentration**
	LC	***Angle* α (°)**	***PS***	***L* (m)**	**N_{pipes}**	**x_{HLSC}**	**y_{HLSC}**	**A_{HLSC} (m²)**	***GWD***	***LR***	**$A_{p,SC}$ (m²)**	***xp***	***yp***	**RA_j**	**(kg m⁻³)**
BC	1	-	-	1000	1	1870	1627	-	1	1	370850	2043	1627	1	1.2×10^{-5}
Fig 4e	2	90	2	1500	2	1871	1463	246263	1	1	706938	2043	1461	1.9	3.0×10^{-5}
Fig 4e_l	2	90	2	1500	2	1871	1463	246263	1	2	724029	2046	1452	2.0	8.2×10^{-3}

Figure supplements [Figure S1 to S4]

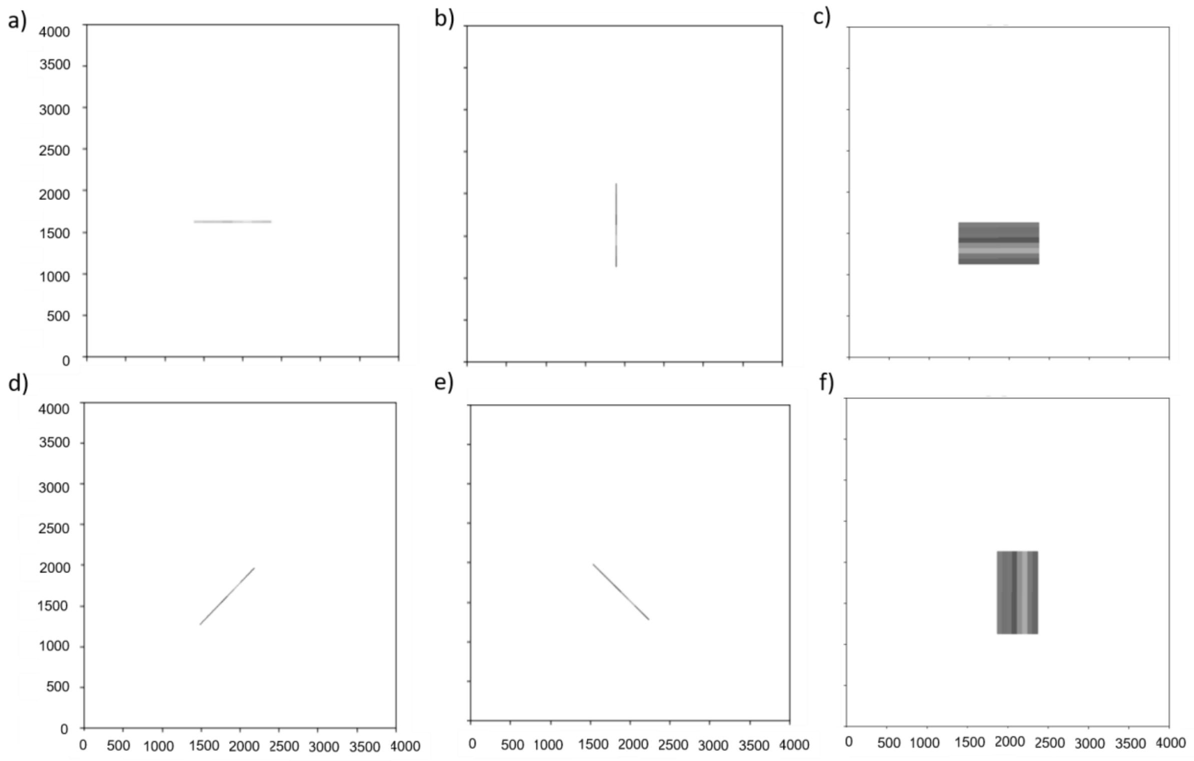

Fig. S1 One HLSC representing one pipe with a constant length of 1,000 m. Level of complexity one: a) width of 5 m, 180°; b) width of 5 m, 90°; d) width of 5 m, 45°; e) width of 5 m, 135°; c) width of 500m, 180°; f) width of 500 m 90°

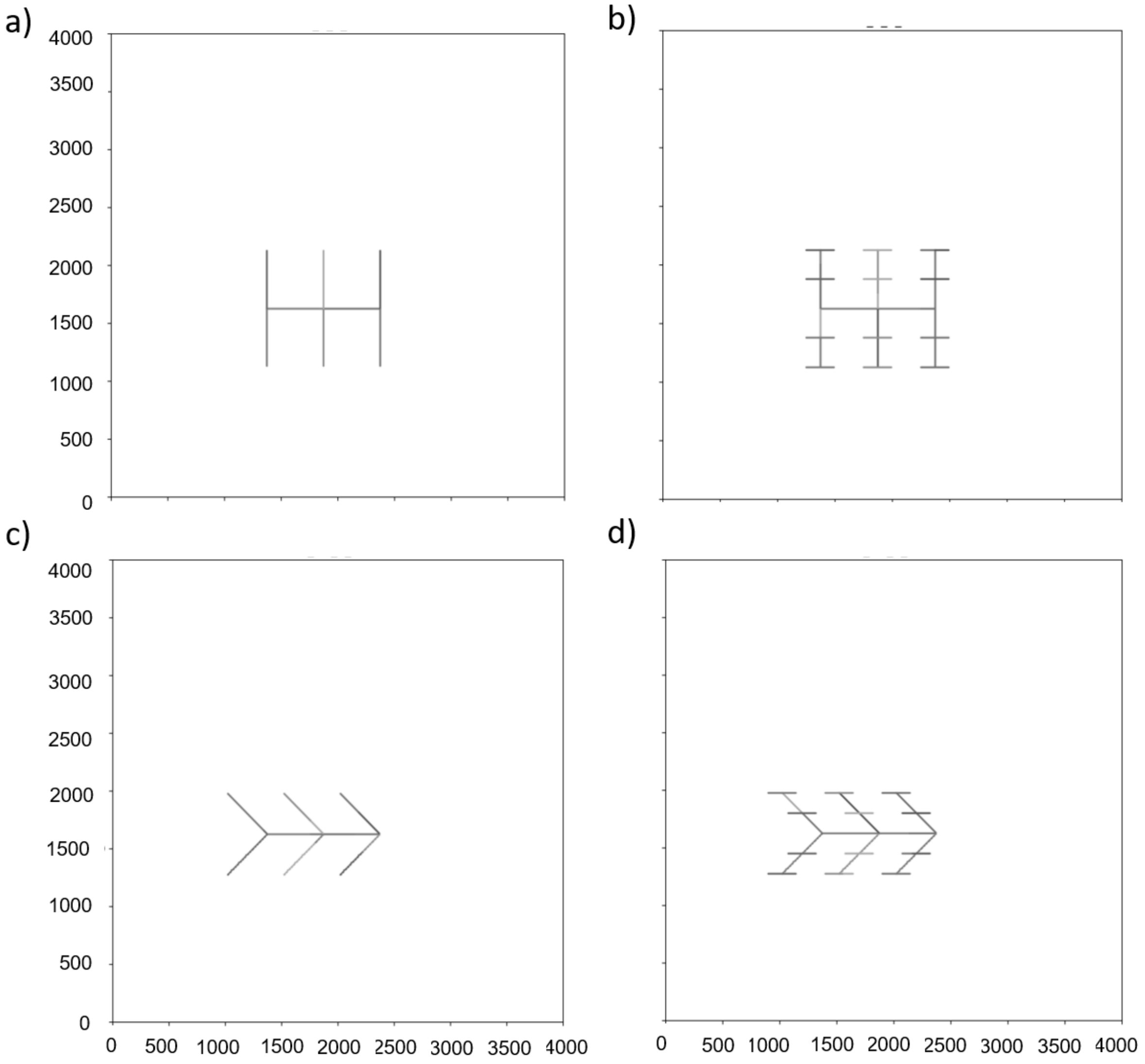

Fig. S2 Examples of the artificial sewer network: a) Level 2 (7 pipes), angle 90°; b) Level 3 (31 pipes), angle 90°; c) Level 2 (7 pipes), angle 45°; d) Level 3 (31 pipes), angle 45°

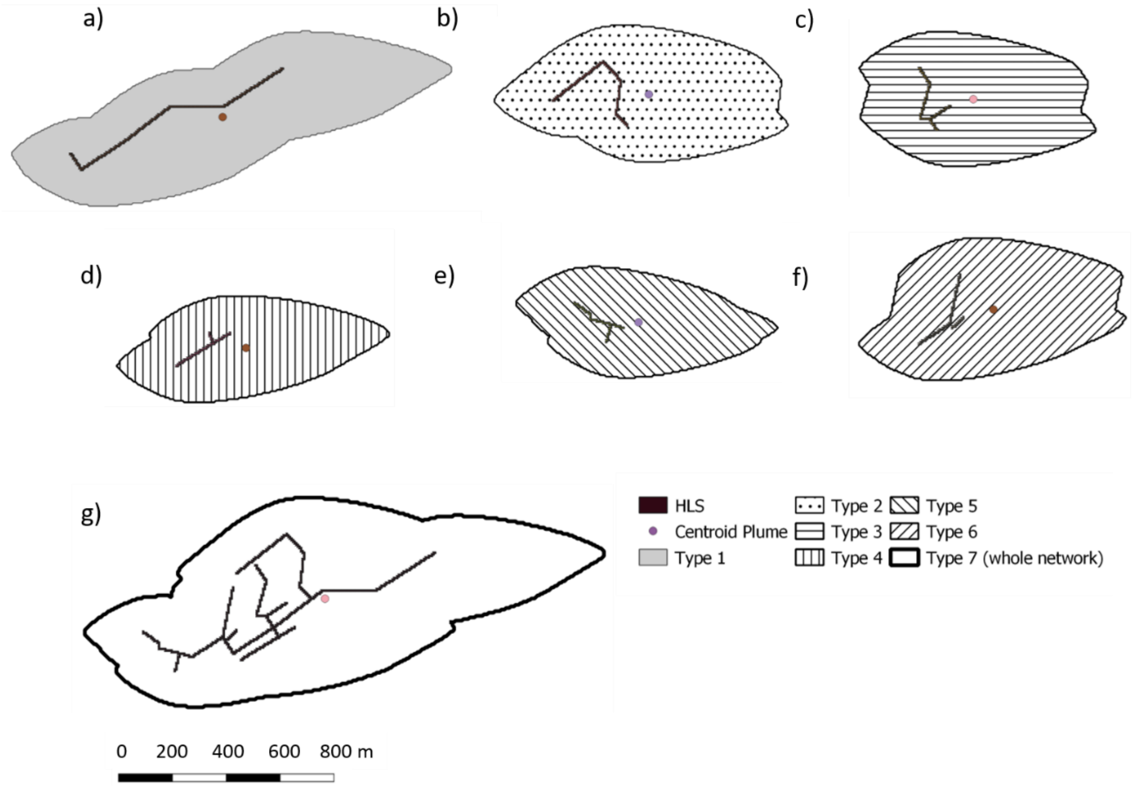

Fig. S3 Edges and covered areas of the contamination plumes generated by each subnetwork (type 1 to 6) and the whole network (type 7) based on an actual UDN in Dresden, Germany

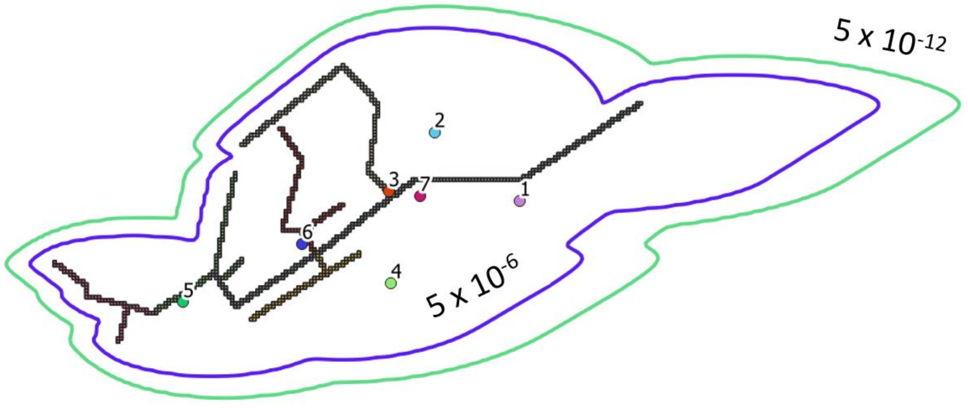

Fig. S4 Edges and covered areas of contamination plume generated by whole subnetwork (type 7) based on a real UDN in Dresden, Germany considering two threshold concentration of contaminant (green line: 5×10^{-12} kg m^{-3} and blue line: 4.6×10^{-6} kg m^{-3}). Locations of geometric centroids of individual plumes generated by HLSC types 1 to 6 are also shown
